# Long noncoding RNAs in the model species *Brachypodium distachyon*

**DOI:** 10.1038/s41598-017-11206-z

**Published:** 2017-09-12

**Authors:** Concetta De Quattro, Mario Enrico Pè, Edoardo Bertolini

**Affiliations:** 1 Institute of Life Sciences, Scuola Superiore Sant’Anna, Piazza Martiri della Libertà 33, 56127 Pisa, Italy; 2 Present Address: Donald Danforth Plant Science Center, 975 North Warson Road, St. Louis, MO 63132 USA

**Keywords:** Gene regulatory networks, Plant genetics

## Abstract

Eukaryotic genomes are pervasively transcribed and only a small portion of the transcribed sequences belongs to protein coding genes. High-throughput sequencing technology contributed to consolidate this perspective, allowing the identification of numerous noncoding RNAs with key roles in biological processes. Long noncoding RNAs (lncRNAs) are transcripts longer than 200 nt with limited phylogenetic conservation, expressed at low levels and characterized by tissue/organ specific expression profiles. Although a large set of lncRNAs has been identified, the functional roles of lncRNAs are only beginning to be recognized and the molecular mechanism of lncRNA-mediated gene regulation remains largely unexplored, particularly in plants where their annotation and characterization are still incomplete. Using public and proprietary poly-(A)^+^ RNA-seq data as well as a collection of full length ESTs from several organs, developmental stages and stress conditions in three *Brachypodium distachyon* inbred lines, we describe the identification and the main features of thousands lncRNAs. Here we provide a genome-wide characterization of lncRNAs, highlighting their intraspecies conservation and describing their expression patterns among several organs/tissues and stress conditions. This work represents a fundamental resource to deepen our knowledge on long noncoding RNAs in C_3_ cereals, allowing the *Brachypodium* community to exploit these results in future research programs.

## Introduction

In the past decade the complexity of eukaryotic transcriptomes, by which genomic regions are largely transcribed into RNAs and give rise to processed and regulated coding and noncoding transcripts, has been revealed^1^. The advent of high-throughput sequencing technologies and computational methods played a crucial role in boosting the annotation of a large number of transcripts, including those rare sequences that could not be detected using other methods, including array-based approaches^2^.

Noncoding RNAs (ncRNAs) are a broad class of molecules, accounting for 90% of the genome, and are ubiquitous components of the transcriptomes^3, 4^. Based on their biological roles, ncRNAs have been divided into: (i) structural ncRNAs (ribosomal RNAs, transfer RNAs, small nuclear RNAs and small nucleolar RNAs) and (ii) regulatory ncRNAs, better known as small ncRNAs (microRNA and small interfering RNAs) and long ncRNAs (lncRNAs)^5^. Although small regulatory RNAs have received large attention in the past decade (miRBase version 21 contains more than 28,000 entries)^6^, lncRNAs have been poorly studied, especially in plants, where few species have been investigated at genomic level so far: *Arabidopsis thaliana*^7, 8^, *Oryza sativa*^9^, *Zea mays*^10, 11^, *Gossypium* ssp^12^, *Populus trichocarpa*^13^ and *Solanum licopersycum*^14^.

LncRNAs are generally defined as a heterogeneous family of long transcripts greater than 200 nucleotides likely transcribed from RNA polymerase (Pol) II and subjected to 5′ capping, 3′ polyadenylation and splicing events, although plant specific Pol IV and Pol V have been also reported to be associated with lncRNAs^15^. In addition, Wu *et al*.^16^ described Pol III transcribed lncRNAs in *Arabidopsis thaliana*. Moreover, even if the main feature of lncRNAs is the lack of evident open reading frames (ORFs), a large fraction of lncRNAs has been shown to be associated with ribosomes in ribosome profiling experiments, showing the potentiality to code for small functional peptides^17^. The features to code for regulatory peptides were also found in the primary transcripts of plant microRNAs, revolutionizing the common idea of coding transcripts^18^. Recently a newly discovered class of endogenous noncoding RNAs, termed circular RNAs (circRNAs), has been described in animal and plant species, adding to the transcriptome complexity^19^.

In general, depending on their genomic origin, lncRNAs are classified in long intergenic ncRNAs (lincRNAs) or long genic ncRNAs, which are further subgrouped in exonic ncRNAs, intronic ncRNAs (incRNAs) and natural antisense transcripts (NATs), transcribed from the complementary DNA strand of the associated coding genes^20, 21^. Because of their mRNA-like features, lincRNAs are the most abundant class of eukaryotic lncRNAs found in poly(A)^+^ RNA-seq data^22^.

The mechanisms of action of long noncoding RNAs are not fully understood yet, but several studies in animals have shown a broad range of action^23^. The spatio-temporal expression patterns linked to the high tissue/organ specificity of these regulatory molecules, together with the ability to regulate gene expression both through *cis* and *trans-acting* mechanisms, make lncRNAs more plastic and capable to act within a broad range of biological pathways^24, 25^.

A large number of lncRNAs were found to be transcribed within the plant genomes^26^ but few clear functional examples of gene regulation mediated by lncRNAs have been described. In *Arabidopsis thaliana*, *COOLAIR* and *COLDAIR* were shown to regulate the expression of the *FLOWERING LOCUS C* (*FLC*) during vernalization^27, 28^. In particular, *COOLAIR*, a natural antisense transcript, causes a transient transcriptional silencing of *FLC* in early cold exposure, whereas *COLDAIR*, an intronic lncRNA, is transcribed from the intronic region of the *FLC* locus and acts repressing FLC expression. This determines a stable change at *FLC* chromatin level through the association with Polycomb Repressive Complex 2 (*PRC2*)^28^. Also in Arabidosis the first example of target mimicry, *INDUCED BY PHOSPHATE STARVATION* *﻿1﻿*(*IPS1*), was discovered and shown to act as a decoy of miR399. This leads to the attenuation of the post-transcriptional repression of the target gene *PHOSPHATE* 2 (*PHO2*)^29^. In cereals, a clear example of lncRNAs implicated in the male fertility is the rice *LONG DAY SPECIFIC MALE FERTILITY ASSOCIATED RNA* (*LDMAR*) that is required for pollen development under long day conditions^30^.

Here we describe for the first time a large catalogue of lncRNAs in *Brachypodium distachyon* (Bd), produced by analyzing public and proprietary transcriptome data sets that include RNA-seq from 26 experiments carried out in the reference inbred lines Bd21, Bd21-3 and in the divergent line Bd1-1. The reliability of our lncRNA discovery pipeline was also independently confirmed by public full-length ESTs. Our catalogue includes 25,338 Bd *bona fide* lncRNAs expressed in various organs and tissues, at different developmental stages and in response to biotic stresses. We investigated lncRNAs expression patterns, highlighting organ, tissue and stress-specific expression profiles. We also discuss the regulatory roles of lncRNA-mediated gene regulation, their potential targets and target mimicry activity.

## Results

### Identification of long noncoding RNAs

Data from twenty-six public and proprietary poly(A)^+^ RNA-seq libraries deposited and available at the National Centre for Biotechnology Information (NCBI) Sequence Read Archive (SRA)^31^ were collected (Supplementary Table 1). The quality of the raw reads was assessed and subjected to adaptors removal and quality filter (see Methods). We retained about 1.5 billion high quality reads (Supplementary Table 2) that were used to re-assemble the transcriptome using a genome guided approach. The various steps of the bioinformatics pipeline used to identify *bona fide* lncRNAs are summarized in the Supplementary Fig. 1. We applied stringent filters based on the main features of lncRNAs currently recognized^22, 32^. By using the program TopHat2^33^ and allowing 2 mismatches we mapped onto the Bd21 reference genome version 2.1^34^ 654 million and 435 million trimmed reads from Bd21 and Bd21-3 respectively. Similarly, 456 millions trimmed reads from the Bd1-1 libraries were aligned to the equivalent re-sequenced genome^35^. In total we aligned 1.39 billion reads using two iteration mapping steps. Transcripts were re-assembled using Cufflinks and an unique transcriptome for each inbred line was generated using Cuffmerge^36^ (Supplementary Fig. 1). Overall 77,016; 59,083; 64,766 transcripts were reconstructed in Bd21, Bd21-3 and Bd1-1 respectively (Supplementary Files 1, 2 and 3). The complete set of 16,079 full length ESTs produced from several Bd21 tissues^37^ was also included in our analysis. These four transcripts data sets were subjected to the pipeline for the lncRNAs identification.

After transcripts reconstruction, the full set of sequences of each inbred line was subjected to six consecutive filters (Supplementary Fig. 1). Concisely, only transcripts longer than 200 bp and encoding ORF smaller than 100 amino acids were kept; transcripts with protein domain annotated in Pfam database and transcripts with low ability to encode proteins were discarded and structural RNAs transcripts were discarded based on sequences similarity with the *Brachypodium* housekeeping RNAs deposited in the Rfam database. The selection steps resulted in a final set of putative lncRNAs: 7,252 in Bd21; 3,715 in Bd21-3; 17,179 in Bd1-1 and 858 in Bd21-EST.

To identify small RNAs associated with lncRNAs we mapped the small RNA sequences available at the plant MPSS database^38^ (http://mpss.udel.edu) and from Bertolini *et al*.^39^ to the four sets of putative lncRNAs (Supplementary Fig. 1). We found on average 5% of lncRNAs associated to small RNAs. Thereby, we classified lncRNAs as *bona fide* lncRNAs that do not share sequence similarity with small noncoding RNAs and lncRNAs associated with small RNAs. Finally, lncRNAs with no counts in all samples were removed from subsequent analyses, resulting in a subset composed by 5,851 in Bd21, 2,681 in Bd21-3 and 15,948 in Bd1-1 (Supplementary Files 4, 5 and 6). We could not apply the selection based on counts level to the 858 lncRNAs found in Bd21-EST (Supplementary File 7), because they were sequenced using the traditional Sanger approach.

To validate the *in silico* lncRNA sequence reconstruction from short reads RNA-seq data we performed a cluster analysis between the lncRNAs found in Bd21 and the complete set of full length Bd21-EST lncRNAs^37^. We found 277 EST sequences (32% of the lncRNAs found in Bd21-EST) clustered with the Bd21 lncRNAs with a 95% sequence identity (Supplementary Fig. 2 and Supplementary File 8). Moreover, we performed RT-PCR amplifications on nine lncRNAs expressed at different level in six Bd21 tissues/organs, such as: third leaf, leaves 20 days, early inflorescence, emerging inflorescence, seed 5 DAP and seed 10 DAP. For all the 9 randomly selected lncRNAs the desired PCR product was obtained (Supplementary Methods).

### Structural and genomic features of long noncoding RNAs

The complete set of long noncoding RNAs identified was examined at sequence and genomic level. We took into account transcripts length, GC content and number of exons in order to highlight possible difference among Bd inbred lines, protein coding genes and plant species.

**Figure 1:**
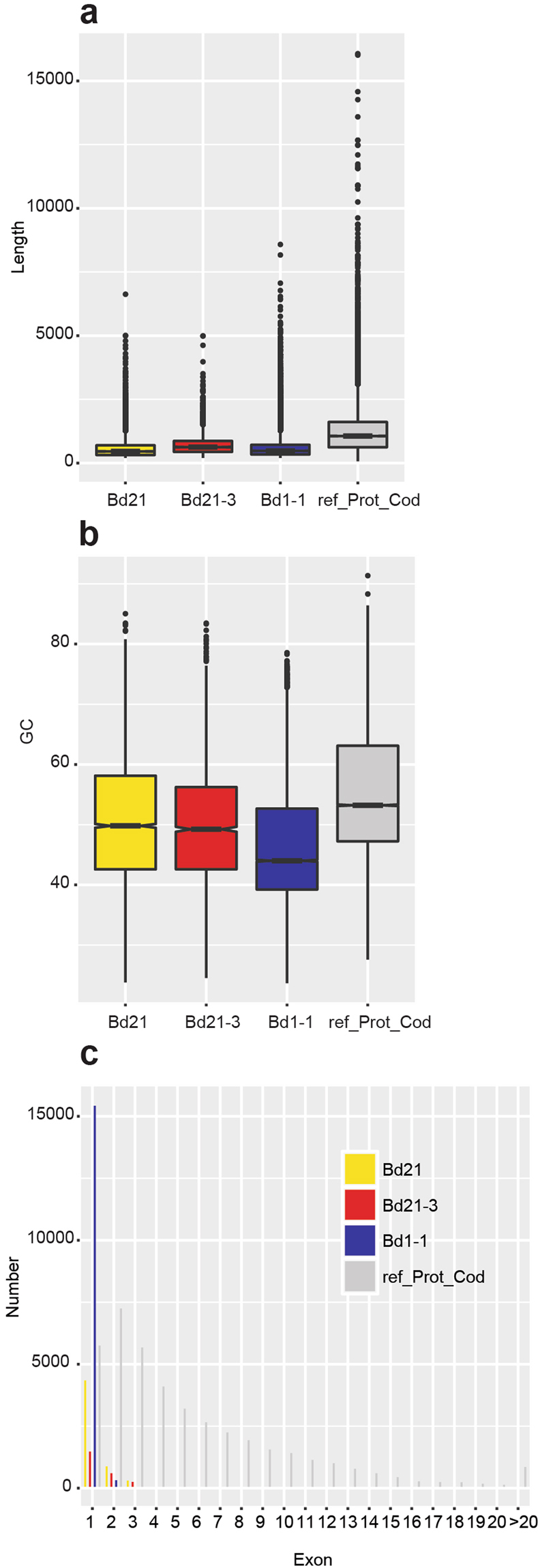
Genomic features of Bd lncRNAs and Bd21 reference protein coding transcripts. The plots show transcript length (**a**), percentage of GC content (**b**) and exons number (**c**) of the lncRNAs annotated in Bd21, Bd21-3 and Bd1-1 and of Bd21 reference protein coding transcripts.

The majority of *Brachypodium* lncRNAs clustered in the range between 200 nt and 700 nt in length (Fig. 1a). The lncRNAs median transcript length in Bd21, Bd21-3, Bd1-1 was respectively 458, 621 and 481 nucleotides, with the first and third quartile ranging between 317 and 868 nt and maximum transcript length of 8,581 nt (Fig. 1a). The GC mean content was between 46% and 50% (Fig. 1b). These features were also confirmed in the pool of lncRNAs identified in Bd21-EST (data not shown). Considering the exons number, a large set of lncRNAs was composed by a single exon with Bd1-1 showing the richest number of monoexonic lncRNAs (97%) (Fig. 1c).

**Figure 2:**
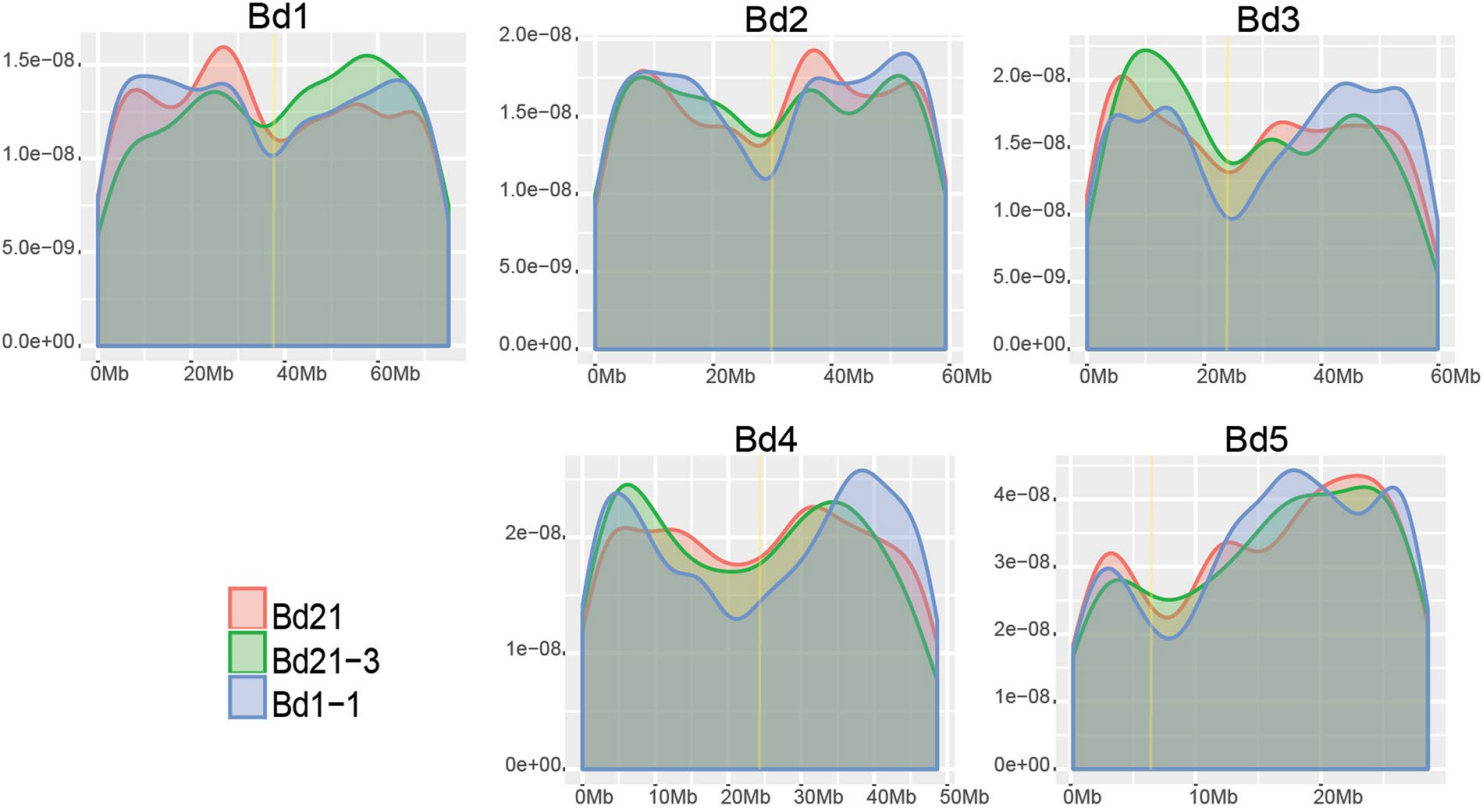
LncRNAs distribution across Bd chromosomes. X axis represents chromosomes length; Y axis represents lncRNAs density. Yellow vertical bar represents the position of the centromeres in Bd21, specifically: 38 Mb in chromosome 1 (submetacentric), 30-31 Mb in chromosome 2 (metacentric), 24 Mb in chromosome 3 (submetacentric), 22 Mb in chromosome 4 (metacentric) and 6 Mb in chromosome 5 (acrocentric) as described by Qi *et al*.^107^.

Differently protein coding mRNAs annotated in Bd21 were characterized by longer sequences with a median of 1062 nt, a higher CG bases composition (mean: 53.63%) and a higher number of exons (Fig. 1). We found lncRNAs equally distributed along the five chromosomes, suggesting a pervasive transcription of the genome and a few lncRNAs clustered in centromeric regions (Fig. 2

).

**Figure 3:**
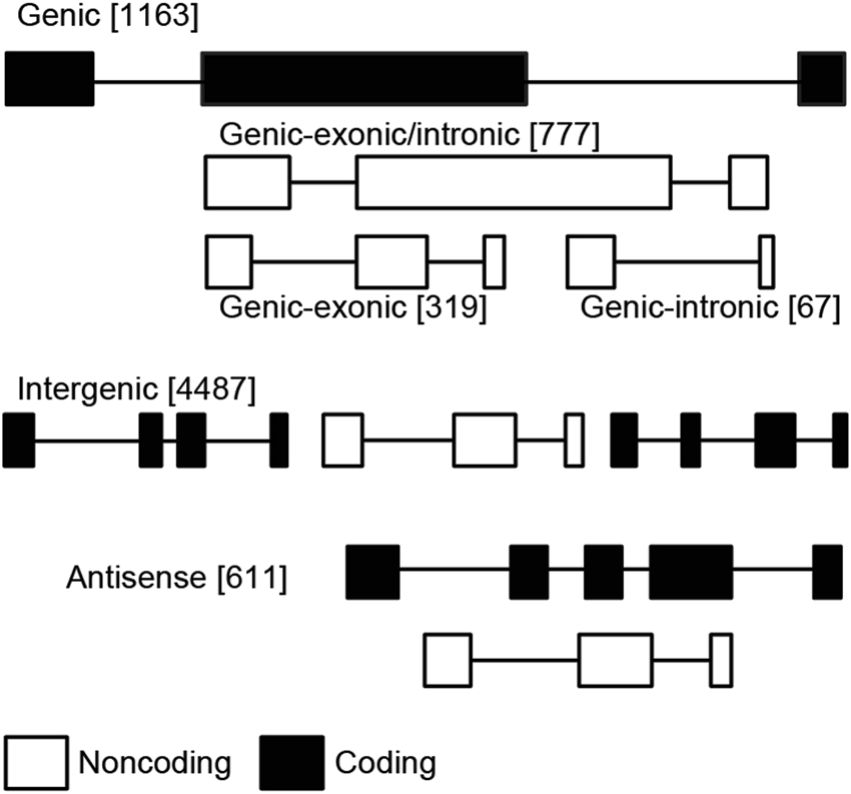
Bd21 lncRNAs genomic classification. Genomic position of lncRNA genes (white bars) in relation to protein-coding genes (black bars). LncRNA genes can be within protein-coding genes (genic lncRNAs) or between two protein-coding genes (intergenic lncRNAs). Genic lncRNAs can be entirely within an exon (genic-exonic) or an intron (genic-intronic) or can span an exonic - intronic region (genic-exonic/intronic). Antisense lncRNAs refer to transcripts that are putatively located in antisense orientation respect protein coding gene.

Focusing on the reference inbred line Bd21, for which a high quality genome assembly and annotation are available, Bd21 lncRNAs were classified according to their genomic location^40^ as long intergenic noncoding RNAs (4,487), exonic lncRNAs (319), intronic lncRNAs (67) and putative antisense lncRNAs (611) (Fig. 3).

### Transposons elements, tandem repeats and microRNA associated with lncRNAs

**Figure 4:**
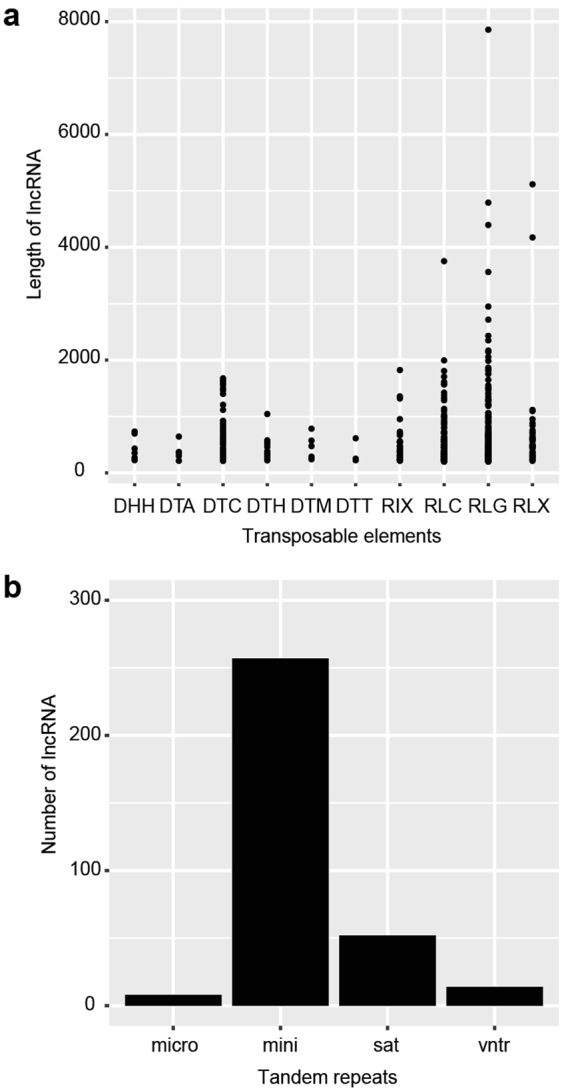
Bd21 lncRNAs associated with transposable elements (TE) and tandem repeats (TR). (**a**) TE and lncRNAs: Y axis is lncRNA length in nucleotides, X axis is TE families. DNA Class II: Helitron (DHH) [7]; Hat (DTA) [5]; CACTA (DTC) [60]; PIF/Harbinger (DTH) [11]; Mutator (DTM) [5]; Tc1/Mariner (DTT) [3]. TE Class I: LINE (RIX) [36], Ty1/copia (RLC) [105], Ty3/gypsy (RLG) [198], Unclassified LTR (RLX) [36]. Round brackets indicate TE code based on the classification system proposed by Wicker *et al*.^42^. Square brackets show number of lncRNA loci associated with TEs. (**b**) TR and lncRNAs: Y axis shows number of lncRNAs, X axis shows types of tandem repeats according to the classification by monomer length into: micro (microsatellites: monomer length 2-9); mini (minisatellites: monomer length 10–99), sat (satellites: monomer length >=100); vntr (variable number tandem repeats: homopolymeric stretches and mixed types).

Recent studies have shown that transposable elements (TEs) permeate long intergenic RNAs^41^. To explore the involvement of TEs in the origin of lncRNAs, we intersected the genomic coordinates of both lncRNAs and annotated TE loci to identify genomic relationship. Based on current TEs classification^42^, we found 466 lncRNAs (7.96%) associated with TE sequences, of which the vast majority (80.47%) belonging to class I retrotransposon. LTRs *Gypsy* (RLG) and LTR *Copia* (RLC) were the most abundant families for class I and CACTA (DTC) for class II (Fig. 4a).

We also investigated the presence of simple tandem repeats (TRs) within lncRNA sequences, by overlapping the current *Brachypodium* TRs annotation with lncRNAs coordinates. We found a positive overlap between 331 minisatellite and lncRNAs (Fig. 4b). To further investigate, at the genomic level the relationship between MIR genes and lncRNAs, we interpolated the coordinates of pre-miRNAs with those of lncRNAs and obtained 8 hits corresponding to the hairpins of miR167c, miR172d, miR395c, miR395j, miR399, miR5059, miR7737 and miR7744. Interestingly, the conserved miR167d and miR399a were found within the intronic regions of the lncRNAs TCONS_00007390 and TCONS_00016338 respectively, suggesting a spliced intron origin from the long noncoding transcripts (Supplementary Fig. 3). Differently, miR395c and miR395j were found within the monoexonic lncRNA TCONS_00072591 (Supplementary File 18), suggesting a simultaneous transcription of a polycistronic transcript. These findings are in agreement with miRNA biogenesis in animals, where miRNAs were discovered in introns and exons of coding or noncoding genes^43^.

### LncRNAs conservation among Brachypodium inbred lines

In this study, we explored the evolutionary conservation of long noncoding RNAs at intraspecific level through sequence similarity searches. Internal lncRNAs redundancy in each inbred line was estimated using the program CD-HIT with a sequence similarity cutoff of 95%. This resulted in 5,698, 2,610, 15,639 uniquely expressed lncRNA in Bd21, Bd21-3 and Bd1-1 respectively (Supplementary Files 9, 10 and 11). A similar cutoff was applied to the lncRNAs BD21-EST data set and we found a high level of sequence redundancy (~50% of the transcript sequences), reflecting cloned mRNA abundance.

LncRNAs conservation at intraspecific level was investigated by searching for sequence homology with a 90% identity among the three Bd inbred lines. We found 135 lncRNAs highly conserved among the three inbreds, showing a sequence conservation that spans almost the entire sequence length. Notably, the majority of these transcripts was found in syntenic and collinear genomic locations (Supplementary Fig. 4).

### Expression profiles of lncRNAs

**Figure 5:**
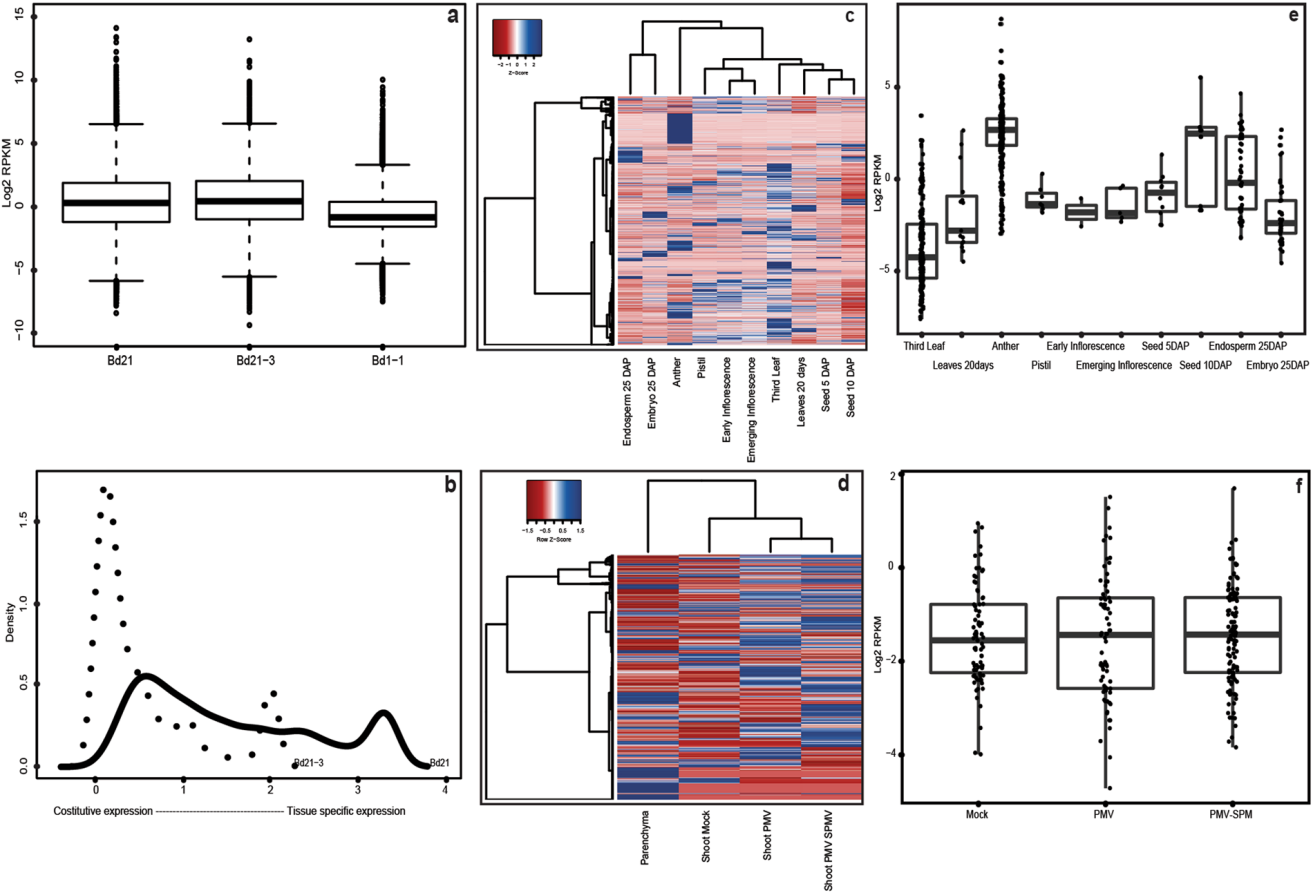
LncRNAs expression profiles in Bd lines. (**a**) Boxplot of lncRNAs expression in Bd21, Bd21-3 and Bd1-1. Expression values are shown as Log2 of RPKM (Reads Per Kilobase per Million mapped reads). LncRNAs not expressed (RPKM = 0) were excluded. (**b**) The Shannon entropy distribution of Bd21 and Bd21-3 lncRNAs. The black line corresponds to Bd21 lncRNAs and the dot line to Bd21-3 lncRNAs. (**c**,**d**) Hierarchical clustering (Ward method) of Bd21 lncRNAs (**c**) and Bd21-3 lncRNAs (**d**) expression profiles. LncRNAs expression levels were scaled to Z score. Blue indicates high expression level, red low expression. (**e**,**f**) Boxplots displaying abundance of specific lncRNAs in Bd21 (**e**) and Bd21-3 (**f**). RPKM expression values are shown as log2 and black dots correspond to individual lncRNA. Parenchyma tissue is not shown in the Bd21-3 boxplot.

We found that Bd lncRNAs were expressed at a low level in all three inbred lines (Fig. 5a). Fifty percent of the lncRNA transcripts showed an expression level greater than 1 read per kilobase per million reads (RPKM) and a maximum expression level at 17,664.4 RPKM (TCONS_00027261) in Bd21 (Fig. 5a). TCONS_00027261 was the most abundant in all Bd21 samples with a minimum TPM of 141.71 and a peak in developing kernels (6957.88 TPM in embryos 25 days after pollination and 3514.14 TPM in endosperm 25 days after pollination) and Pistil (5420.72 TPM) (Supplementary File 19).

To better explore the complexity of the lncRNA transcripts found we did not apply any further filter based on RPKM values since lncRNAs have been shown to be low expressed, extremely heterogeneous in their expression patterns and with short half-life^7, 9, 11^. In addition, due to the intrinsic nature of lncRNAs, it was shown that large data sets need to be investigated in order to capture the complete constitutive and tissue specific expression of the noncoding transcriptome^44^. Moreover, some works used a low expression threshold, e.g. 0.3 RPKM to define genes constitutively expressed^45^. In our data set lncRNAs with RPKM <0.3 were 301, 394 and 3,218 in Bd21, Bd21-3 and Bd1-1 respectively. In addition some lncRNAs with expression level <1 RPKM were also retrieved in the Sanger EST collection (see Methods).

Tissue/organ specific expression was assessed in Bd21 and Bd21-3 by computing the Shannon Entropy^46, 47^ (see Methods). LncRNAs were generally found uniformly expressed in all samples (entropy = 0) but, on average, 10% were specifically detected in only one tissue/organ (entropy >3 in Bd21 and entropy = 2 in Bd21-3) (Fig. 5b).

Overall, the expression levels of lncRNAs vary significantly in the different samples, from ubiquitous (837 in Bd21) to tissue/organ specific (590 in Bd21), showing divergent and specific degrees of expression (Fig. 5c,d). In Bd21 the highest number of specificity was observed in anther (44%), in the third growing leaf (32%) and in the endosperm 25 days after pollination (9.76%) (Fig. 5c and Supplementary Fig. 5). Interestingly, we found that lncRNAs specifically expressed in anthers (254) had the highest expression (Fig. 5e).

Concerning Bd21-3 line, shoot samples infected with *Panicum mosaic virus* (PMV) and its satellite virus (SPMV)^48^ showed the strong modulation of lncRNA expression during the plant-virus interactions (Fig. 5d). This result highlighted a core set of 1,743 lncRNAs constitutively expressed and a treatment specific set expressed in response to the pathogen infection (78 Mock specific, 73 PMV specific and 121 PMV + SPMV specific) (Fig. 5f and Supplementary Fig. 5).

### Interactions between microRNAs and long noncoding RNAs

To understand the link between microRNAs and lncRNAs in *Brachypodium distachyon* we investigated the crosstalk between lncRNAs and microRNAs by identifying lncRNAs targeted by miRNAs and lncRNAs acting as miRNA decoy.

**Figure 6:**
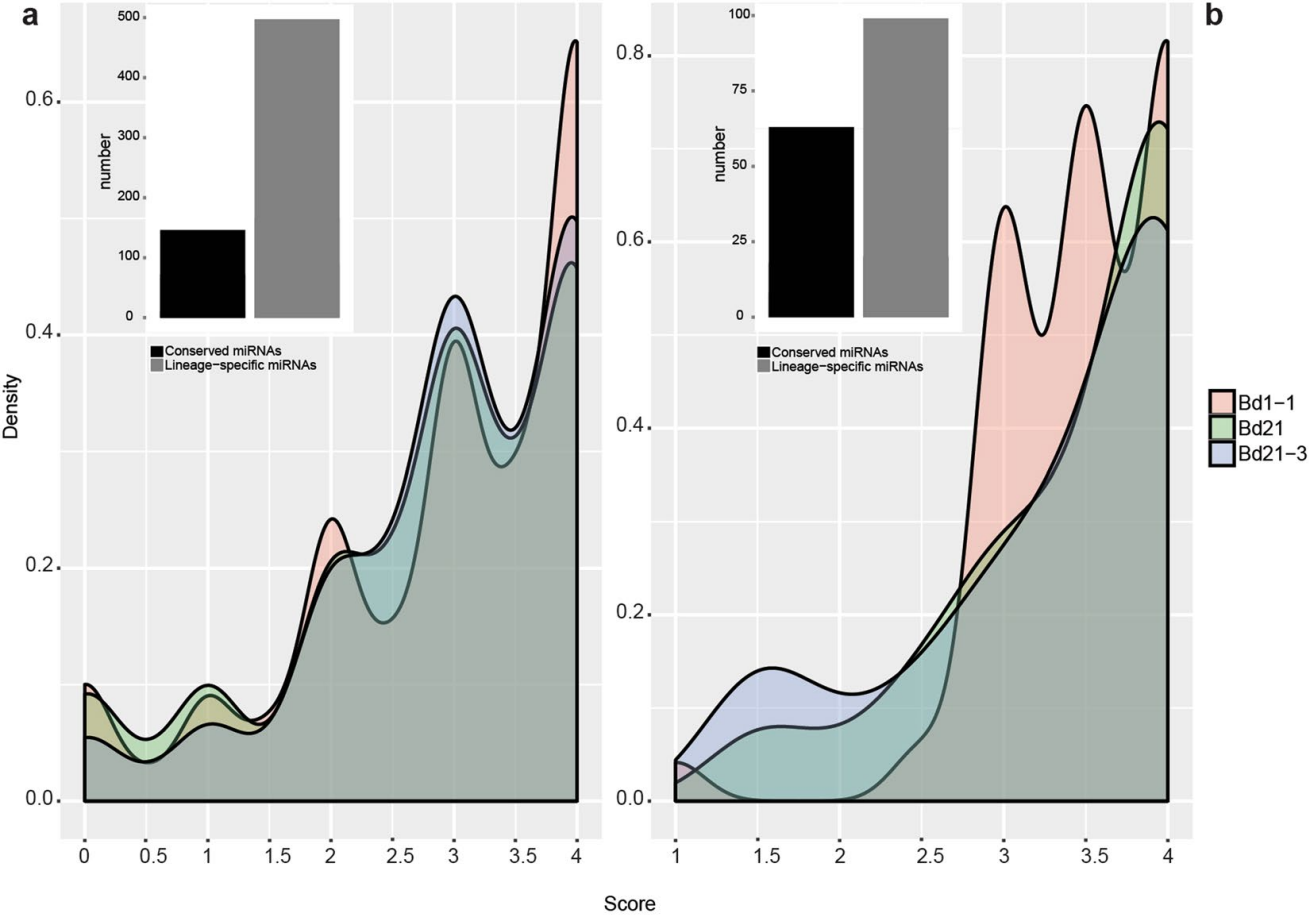
Score values resulting from *in silico* prediction of lncRNAs target of microRNAs and lncRNAs acting as target mimicry. (**a**) Distribution of scores obtained from TargetFinder analyses of lncRNAs identified as target of microRNAs. The bar-plot shows the number of conserved and lineage-specific microRNAs Bd families predicted by TargetFinder with a score 2. (**b**) Score of lncRNAs identified as sponge of microRNAs by psMimic. The bar-plot shows the number of conserved and lineage-specific Bd microRNAs families regulated by lncRNAs. In both density plots lines correspond to each of the inbred line Bd21, Bd21-3 and Bd1-1.

We identified miRNAs able to target lncRNAs using the program TargetFinder^49^. A total of 3,753 targets were retrieved. Among these, 25% of the predictions had a target score ≤2 with an almost perfect sequence complementarity, resembling *bona fide* targets (Fig. 6a). Notably, within this subgroup of targets the miRNAs mostly belong to lineage-specific miRNA families (miR1122, miR1135, miR5171, miR5174, miR5175, miR5180, miR5181, miR5183, miR5185, miR7758, miR9493) with the three families miR1122, miR5174 and miR5181 that account for the vast majority of putative lncRNA targets that are characterized by a high degree of conservation in the first 10 nucleotides at the 5′ end of the miRNA target site (Supplementary Figs 6–8). MiR5174 and miR5181 were previously found to originate from repetitive regions rich in heterochromatic 24 nt small RNAs^50^ whereas miR1122 was found associated with biotic and abiotic stresses in wheat and barley^51–53^.

**Figure 7:**
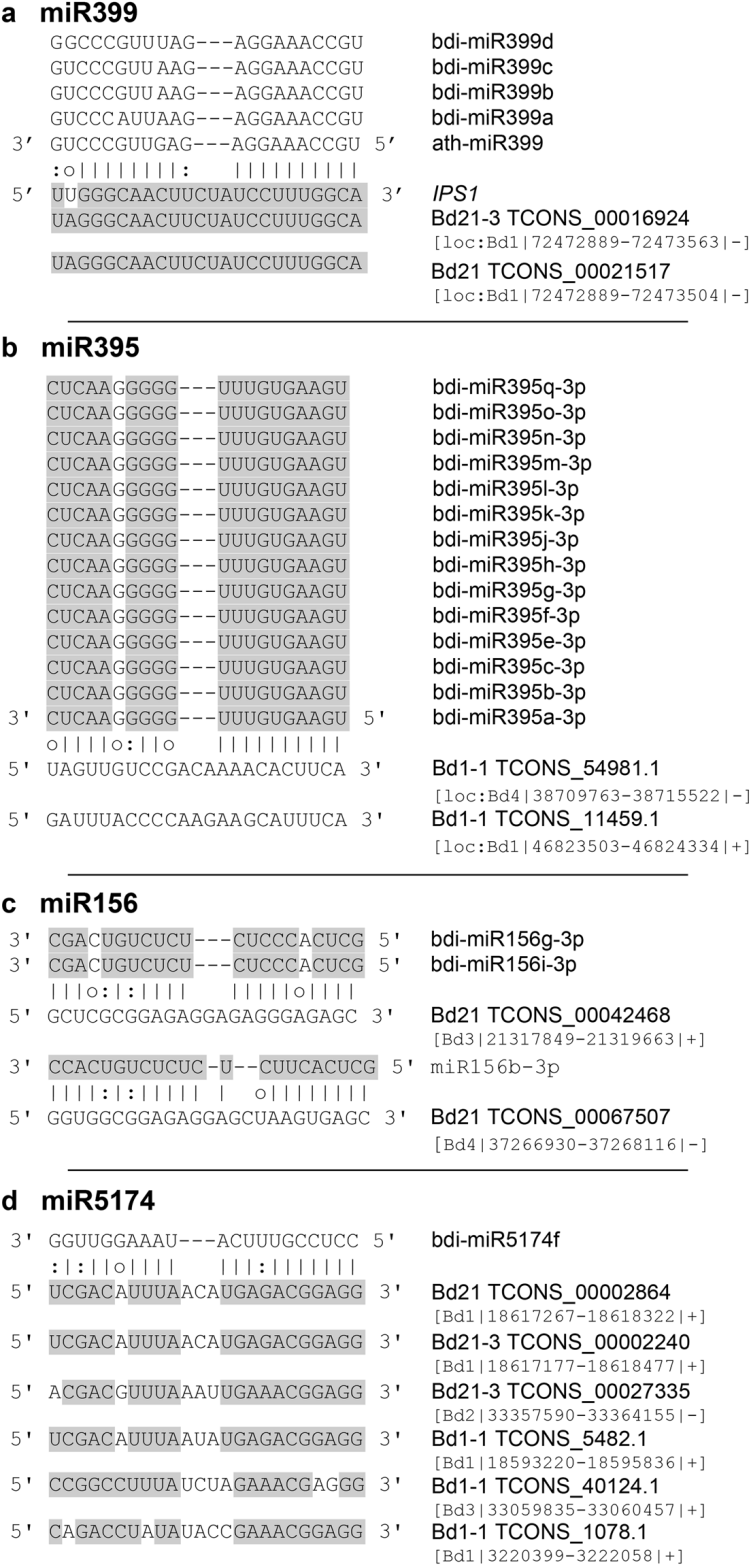
*In silico* endogenous target mimics and their corresponding microRNAs. (**a**) Bd lncRNAs target mimic of miR399. The figure shows the conservation of the target mimic binding site between Bd and Arabidopsis *IPS1* lncRNA. (**b**) Bd lncRNAs target mimic of miR395. (**c**) Bd lncRNAs target mimic of miR156. (**d**) Bd lncRNAs target mimic of miR5174. Grey regions show sequence complementarity between microRNAs and lncRNAs sequences. The dash line indicates the bulge between lncRNA and microRNA molecules. The genomic coordinates are provided next to each lncRNA.

Target mimicry (TM) lncRNAs were predicted using the algorithm psMimic^54^, that led to the identification of 228 TM hits with complementary score ranging between 0 and 4. The majority of miRNA sequences retrieved in this screening belonged to lineage-specific miRNAs and was characterized by the typical central-nucleotide bulge/mismatch surrounding the 10^th^–11^th^ nucleotides of the miRNA (Fig. 6b), which is the canonical cleavage site catalyzed by *ARGONAUTE*^55^. We also found TMs related to large miRNA families such as miR156, miR395, miR399 and miR5174. LncRNAs TCONS_00021517 and TCONS_00016924 have sequence complementarity in the TM site with the mature sequence of miR399a,b,c,d (Fig. 7a). miR399 is involved in the phosphate homeostasis and takes part in the systemic signaling pathways to communicate phosphorus availability and demand between shoot and root^56, 57^. In *A*. *thaliana* the lncRNA *IPS*1 (*INDUCED BY PHOSPHATE STARVATION 1*) was found to actively attenuate the expression of miR399, mediating a TM activity and increasing the expression of the phosphate homeostasis target gene *PHOSPHATE* 2 (*PHO2*)^29^. Although we did not find any sequence homology between the *B*. *distachyon* lncRNAs (TCONS_00021517 and TCONS_00016924) and *A*. *thaliana IPS1* along the entire transcript we observed strong conservation in the TM site sequence. Moreover, miR395, which is involved in the regulatory network of sulfate assimilation^58^, was found in our Bd1-1 data set associated through a TM mechanism with the two lncRNAs: TCONS_54981.1 and TCONS_11459.1 (Fig. 7b).

MiR156, which plays a role in controlling flowering and leaf development^59^ appeared targeted by several lncRNAs. In particular, the 3′ of miR156g,i was found to be potentially targeted by the TM lncRNA TCONS_00042468 that is highly expressed in Bd21 early inflorescence (44.6579 TPM) and pistil (32.062 TPM). A similar pattern of expression has been highlighted for TCONS_00067507 which shows sequence complementarity with bdi-miR156b-3p (Fig. 7c). This computational approach allowed us to identify a similar TM Bd21-3 and Bd1-1 likely to bind both bdi-miR156b,g,i-3p and bdi-miR156a,j-5p, and bdi-miR156d,h-3p, respectively (Supplementary Files 16, 17). Similarly, the lineage-specific miR5174f was found associated with a perfect complementarity in the TM region to several lncRNA transcripts that might act to sequestrate the mature miRNA (Fig. 7d).

## Discussion

In recent years an increasing number of studies has revealed the complexity of the eukaryotic transcriptome and the important role of noncoding RNAs in regulating gene expression^60^. This complexity has been deeply investigated in animal models and it is now emerging also in plant species due to the advent of high-throughput technology and the extensive application of RNA-seq approaches^61–63^.

In this work we conducted an extensive annotation of lncRNAs in *Brachypodium distachyon*, using the data from public and proprietary poly(A)^+^ RNA-seq experiments produced from several tissues/organs in different Bd lines. Overall our study led to the identification of 25,338 *bona fide* lncRNAs in the genotypes 21, 21-3 and 1-1.

Moreover, due to the intrinsic limit of the poly(A)^+^ RNA-seq libraries, in our study we could not identify lncRNAs transcribed by Pol III, IV, V since they do not contain a poly(A) tail and are mostly enriched in ribo-minus RNA-seq experiments^64^. Bd lncRNAs were characterized by a median length ranging between 458–621 bp and were mostly monoexonic transcripts. Among the lncRNAs here identified, the large majority was classified as long intergenic RNAs, confirming lincRNA as the most abundant class of long noncoding genes present in poly(A)^+^ RNA-seq experiments^21, 22^.

Sequencing depth of RNA-seq and fragmented genome assembly lead to the identification of a large number of lncRNAs^44, 65^. Thereby, mRNA-seq depth in Bd1-1 and the current draft version of its reference genome^35^ could in part explain the higher number of lncRNAs found in this divergent inbred line.

According to their functional importance, lncRNAs are expected to be conserved at different levels: sequence, structure, function and expression profiles^66^. Although at primary sequence level lncRNAs showed a low conservation^67^, other lncRNAs features such as syntenic relationships, microhomology and secondary structure resulted in higher degree of conservation^65, 68^.

Investigation at primary sequence level among inbred lines suggests a low intraspecific lncRNAs conservation. In fact, although Bd21 and Bd21-3 lines were expected to be phylogenetically closed, only 47% of lncRNA sequences showed a high degree of sequence conservation. Natural diversity study based on SSR markers showed Bd1-1 as the most divergent line within the Bd genotypes, whereas Bd21-3 was considered genetically distinct from Bd21 even if both originated in the same location site^69^. Moreover, current knowledge suggests that lncRNAs diverge rapidly, being subjected to different selective pressure as compared to protein coding genes, accumulating base substitutions and indels. A possible explanation for the lack of extensive conservation is that most lncRNAs could be subjected to exaptive instead of adaptive evolution^70^.

In vertebrates, transposable elements and tandem repeats elements were shown to be strongly enriched in lncRNAs. This suggests their role in the origin of lineage-specific lncRNAs^71^. In addition, human TEs, mostly LTR retrotransposons class, were found to pervade lincRNAs, suggesting a role in controlling lncRNAs transcription and specific expression^41, 72^. *Brachypodium* genome is characterized by a relatively low number of retrotransposons^34^, and class I TEs is principally composed by *Copia* and *Gypsy* elements. Indeed, in our data set we retrieved Bd21 lncRNAs that were mainly associated to *Copia*/*Gipsy* LTR and to *CACTA* DNA transposons, which have been also found abundant in many *Triticeae* species where they are involved in gene regulation^73^. In this frame, our results could support the novel hypothesis named Repeat Insertion Domains of LncRNAs (RIDL)^74^, according to which TE-derived fragments of lncRNA act as structural and regulatory domains. Tandem repeats (TRs) occur frequently in many eukaryotic genomes where they are interspersed in different genomic locations, including coding and noncoding genes^75^. In humans, TRs have been found enriched in genes and regulatory regions participating in transcriptional regulation and development processes^76^. Although a few studies in plants have shown the highest density of TRs in 5′-UTRs, promoters and intergenic regions^77, 78^, the link between TRs and lncRNAs has not been clearly examined so far. Our work shows the strong association of lncRNAs with minisatellite TR.

Higher eukaryote transcriptomes are diverse and dynamic, with lncRNA loci exceeding those of mRNAs and exhibiting specificity, context and time dependent expression, a shorter half-life and a low expression^79, 80^. We found all those features in the Bd lncRNAs we identified. Bd lncRNA transcripts showed lower abundance than protein coding genes, even though some lncRNAs exhibited extremely high, dynamic and specific expression profiles. Bd male gametophyte was characterized by the greatest number of specific lncRNAs, similarly to rice and maize^9, 11^. Phased small-interfering RNAs (phasiRNAs) originated from noncoding RNA precursors were found abundant in panicle of several grass species, including *Brachypodium*^34, 81, 82^. Accordingly, in Bd the expression pattern of anther specific lncRNAs might correlate with the presence of phased loci, being part of the phasiRNA biogenesis machinery in which the synthesis requires both miRNAs and phasiRNA precursors. In particular, Zhai *et al*.^83^ highlighted the synthesis of miR2118 dependent 21 nt and miR2275 dependent 24 nt phasiRNAs, putatively implicated as mobile signals in anther development, coordinating cell-type specific expression^84^. It is interesting to point out the analogy that noncoding RNAs are involved also during spermatogenesis in several animal models systems, including mouse, where regulatory RNAs such as microRNAs, siRNAs, Piwi-interacting RNAs and long noncoding RNAs participate in the strict developmental process giving rise to mature spermatozoa^65, 85^.

MicroRNAs (miRNAs) have been clearly shown to act as post-transcriptional regulators of gene expression, whereas long noncoding RNAs only recently emerged as new regulatory molecules involved in several biological pathways using a plethora of mechanisms^86, 87^. In plant and animal systems the influence of lncRNAs upon microRNAs, termed target mimicry (TM) or competing endogenous RNA (ceRNA), has been reported in several works^9, 29, 54, 88, 89^ that showed the ability of noncoding transcripts to serve as endogenous sponge able to sequestrate miRNAs. Differently, the influence of microRNAs on gene expression through the targeting of long noncoding RNAs via complementary sequence site is now only emerging^90^.

The first evidence of the TM was observed in *Arabidopsis thaliana*, where miR399 is efficiently modulated by *IPS1* harboring a complementary sequence site to miR399^29^. This characteristic, highlighted in other TM lncRNAs, led us to assume that the regulatory mechanism controlling miR399 through TM mechanism could be conserved between dicot and monocot species^54, 91^. Moreover, the polycistronic miR395 family, involved in the sulfate assimilation^58^, was found in our Bd1-1 data set associated through a TM mechanism with two lncRNAs. Clustered miRNAs have been shown to be transcribed into polycistronic transcripts encoding homologous miRNAs. In this context, the presence of lncRNAs with miRNA target mimic pairing could potentially compete with the RISC-complex, sequestering the entire miR395 cluster.

Here for the first time we generated a collection of *in silico* predicted target mimic lncRNAs that are a valuable starting point to investigate miRNA repression based on miRNA target mimic. In general, we observed that the majority of TM interactions is between lineage-specific miRNAs and lncRNAs, suggesting a convergent evolution of the entire regulatory RNA noncoding component of *Brachypodium* for the control of specific biological processes.

Accordingly, the interaction of lncRNAs, miRNAs and their direct mRNA targets might be linked in regulatory nodes (lncRNA-miRNA-targets) that coordinate gene expression programs at specific developmental stages. With this genome-wide characterization of the *Brachypodium* lncRNAs component we provide novel clues and tools to speed up the identification and validation of RNA based regulatory nodes in grasses that could be useful targets for biotechnology applications.

## Methods

### Data sets used in this study

This work is based on the data produced in several RNA-seq experiments carried out in the three *Brachypodium distachyon* inbred lines Bd21, Bd21-3 and Bd1-1, whose list is reported in the Supplementary Table 1. All data were downloaded from the National Centre for Biotechnology Information (NCBI) Sequence Read Archive (SRA)^31^. In addition, we included the data from a proprietary RNA-seq library produced from a pool of third leaves in the reference inbred Bd21. As reported in Supplementary Table 1, the public Bd21, Bd21-3 and Bd1-1 poly(A)^+^ RNA-seq data include several tissues and organs. Specifically, the Bd21 data set from Davidson *et al*.^92^ comprises leaf collected at 20 days after sowing, early and emerging inflorescence, pistil, anther, seed at 5 and 10 days after pollination (DAP), embryo at 25 DAP, endosperm at 25 DAP. The Bd21-3 data set includes two experiments consisting in: pooled libraries of parenchymatic tissue and shoots plants collected 7 days post infection with *Panicum mosaic virus*^48^. The Bd1-1 data set comprises a pooled RNA-seq experiment produced from third leaves harvested from 3-week-old plants^35^. Within the three data set, the samples of leaf collected 20 days after sowing and embryo at 25 DAP included in Bd21 data set have two biological replicates. While each library produced from Bd21-3 infected plants included three mixed biological replicates. We included also full length ESTs dataset derived from several Bd21 tissues/organs and treatments (seed at germination, leaf at vegetative stage and after flowering, shoot, crown, spikes at flowering and at different stages after pollination, callus and leaf at 2 weeks after germination treated with different stresses and compounds)^37^ to provide an independent validation to our computational pipelines for lncRNA discovery and to increase our catalogue of Brachypodium lncRNAs. We used the Bd21 version 2.1 genome and Bd1-1 re-sequenced genome, downloaded from Phytozome version 10 (https://phytozome.jgi.doe.gov) as reference genome sequences.

### Plant material

Bd21 third leaves were grown as previously described by Verelst *et al*.^93^. cDNA libraries were prepared from three independent experiments, and each experiment consisted of a collection of 400 plants. Third leaves were collected after 24 hours from their emergence, stored in RNA*later* solution (Thermo Fisher Scientific) and successively frozen in liquid nitrogen. Total RNA samples were extracted using the Plant/Fungi total RNA purification kit (Cat. 25800) from Norgen Biotek Corp. Poly(A)^+^ RNA-seq libraries were produced according to the Illumina TruSeq RNA library preparation kit and sequenced (50 bp single-read) using Illumina HiSeq 2000. Data are available on SRA BioProject PRJNA386608.

### Transcriptome reconstruction

Raw reads were processed by removing the sequencing adapters using the program Cutadapt version 1.2.1^94^ and by filtering low quality reads (Phred score ≥30) with ERNE-FILTER version 1.3^95^. Trimmed reads from the Bd21 and Bd21-3 were mapped against the reference Bd21 genome (version 2.1), whereas reads from the inbred line Bd1-1 were mapped against the re-sequenced genome (version 1)^35^, using the spliced aligner TopHat^33^ version 2.0.9. To exploit the ability of using splice site information derived from the first alignments, a second round of mapping was carried as suggested by Cabili *et al*.^96^. For each library, the transcriptome was independently re-assembled using Cufflinks^36^ version 2.0.9. Subsequently we used Cuffmerge to obtain a unique non-redundant transcriptome for each inbred line. Finally, the reconstructed transcripts sequences were retrieved using the gffread tool.

### LncRNAs identification pipeline

LncRNAs were filtered out from the entire collection of assembled transcripts by applying a stringent stepwise filtering pipeline based on the currently established lncRNAs features^22, 32^. Our pipeline described in De Quattro *et al*.^97^ is composed by six consecutive filters: (i) a size cutoff based on the assumption that lncRNAs are longer than 200 nucleotides; (ii) an *open reading frame* (ORF) putatively coding for a peptide sequence shorter than 100 amino acids. Since lncRNAs can code for small peptides^98^, this selection allows to retain a significant level of stringency without losing many potential lncRNAs; (iii) known protein domain identification using protein sequences downloaded from the Pfam database^99^ version 27 (BlastX with E-value ≤ 0.001) to eliminate transcripts encoding protein; (iv) a *coding potential calculator* (CPC) to test the protein coding potential of the remaining transcripts^100^; (v) housekeeping noncoding *RNAs* to exclude all transcripts homologous to *Brachypodium* genomic and plastidial tRNA, rRNA, snRNA and snoRNA retrieved from the Rfam database^101^ version 12 (http://rfam.xfam.org/) and (vi) the precursor of small noncoding RNAs to filter out transcripts with noncoding features associated to small RNAs. For this final step of the pipeline, nineteen Bd21 small RNA-seq data from several tissues, organs and treatments were downloaded from the Plant MPSS database^38^, plus eight Bd21 small RNA-Seq libraries produced in our laboratory from young developing leaves^39^. *Bona fide* lncRNA identified in Bd21, Bd21-3 and Bd1-1 were named using the prefix *TCONS*.

### Validation of Bd21 lncRNA transcripts

The quality of our *in silico* lncRNA transcripts was assessed using as benchmark ~16,000 full length ESTs generated by Sanger technology^37^. The CD-HIT^108^ program version 4.6 was applied to perform a cluster sequence analysis to check sequence identity between lncRNAs and ESTs with a cutoff equal to 95%. Results were further investigated using Dotter^102^ and mVISTA^103^.

### Classification of lncRNAs

LncRNAs were classified into four main categories based on their location relative to protein coding genes: (i) intergenic; (ii) genic-intronic; (iii) genic-exonic and (iv) putative antisense lncRNAs, on the condition that the sequence was entirely contained within the above classes. LncRNAs classification was conducted using the Bioconductor package GenomicFeatures^104^. Genic, exonic, intronic and intergenic regions were selected from the Bd21 annotation version 2.1. To identify antisense lncRNA we used the approach described in Li *et al*.^11^.

### LncRNAs associated with transposable elements, tandem repeats and microRNAs

Transposable elements (TEs) associated with the lncRNA loci were determined based on the current Bd21 TEs annotations, retrieved from MIPS (ftp://ftpmips.helmholtz-muenchen.de/plants/brachypodium/repeats). LncRNAs coordinates were intersected with TEs coordinates using the Bioconductor package GenomicFeatures^104^. We retained only lncRNAs contained within the TEs genomic coordinates. In addition, we also investigated the presence of tandem repeats (TRs) and precursors of microRNAs within the putative lncRNA sequences.

### Sequence similarity analysis

We performed a clustering analysis to assess lncRNA sequence similarity among the three inbred lines using CD-HIT version 4.6. LncRNA sequences of each inbred line were first clustered separately, considering a cutoff of 95% to reduce sequence redundancy. Unique lncRNA sequences in each inbred line were then compared pairwise, with the threshold of sequences nucleotides similarity set at 90%.

### Expression level and tissue specificity of lncRNAs

Two normalization methods were used to investigate the lncRNAs expression level: (i) RPKM (Reads Per Kilobase per Million mapped reads) for the intra-sample quantification, by counting reads mapped on the reconstructed transcript models with the script htseq-count^109^ version 0.5.4p5 (with option intersection-noempty to exclude multi-mapping reads). The resulting matrix of counts was used to discard transcripts not expressed (those with zero counts in all samples), and the expression level was quantified in RPKM using the Bioconductor package edgeR^105^. (ii) TPM (Transcripts Per Million) for inter-sample quantification, by using the program Salmon^106^ in the quasi-mapping based mode. Specific expression of lncRNAs was determined using the Shannon Entropy method with the BioQC package from Bioconductor.

### Identification of lncRNA targets of microRNAs and competing endogenous target mimics

We searched for the lncRNAs as potential target of microRNAs and lncRNAs target mimic activity. We collected *B*. *distachyon* miRNA mature sequences from the miRBase version 21. TargetFinder (https://github.com/carringtonlab/TargetFinder) with default options was used to identify lncRNAs potentially targeted by miRNAs, applying default parameters. According to the TargetFinder score based criterion^49^ we considered potential target of miRNAs all the lncRNAs that in the analysis have with a cutoff ≤4.

Competing endogenous target mimics (eTMs) activity was determined by locally running the software psMimic^54^ version 1.1 with default parameters (http://omicslab.genetics.ac.cn/psMimic).

### Data availability

All supplementary files are available at Figshare https://doi.org/10.6084/m9.figshare.3423635.v1 through the link https://figshare.com/s/35435039f2ea3d7bb4ef.

## Electronic supplementary material

Supplementary information
