# Publisher Correction: Long noncoding RNAs in the model species *Brachypodium distachyon*

**DOI:** 10.1038/s41598-020-67058-7

**Published:** 2020-06-23

**Authors:** Concetta De Quattro, Mario Enrico Pè, Edoardo Bertolini

**Affiliations:** 1 Institute of Life Sciences, Scuola Superiore Sant’Anna, Piazza Martiri della Libertà 33, 56127 Pisa, Italy; 2 Present Address: Donald Danforth Plant Science Center, 975 North Warson Road, St. Louis, MO 63132 USA

Correction to: *Scientific Reports* 10.1038/s41598-017-11206-z, published online 12 September 2017

In the original version of this Article, the authors Concetta De Quattro and Mario Enrico Pè were incorrectly indexed. This error has now been corrected.

