# Supplementary material for: Long noncoding RNAs in the model species Brachypodium distachyon
Source: Sci Rep. 2017 Sep 12;7:11252. doi: 10.1038/s41598-017-11206-z (PMC5595811; doi:10.1038/s41598-017-11206-z)
Supplement: Supplementary file 1 — Supplementary information [file 41598_2017_11206_MOESM1_ESM.pdf]

Long noncoding RNAs in the model species *Brachypodium distachyon*

Concetta De Quattro, Mario Enrico Pè, Edoardo Bertolini

Supplemental Figure 1:
In silico workflow describing transcriptome reconstruction and lncRNAs identification.
(A) Analysis of RNA-seq libraries leading to transcriptome reconstruction.
(B) The bioinformatic pipeline used to annotate lncRNAs in each inbred line. Bd21 full length EST database was also considered. (C) Number of potential lncRNAs identified in each inbred line. Potential lncRNAs were aligned to Bd21 small RNAs by Patman to identify sRNAs associated to lncRNAs. (D) Number of bona fide lncRNAs and small RNAs associated to lncRNAs.

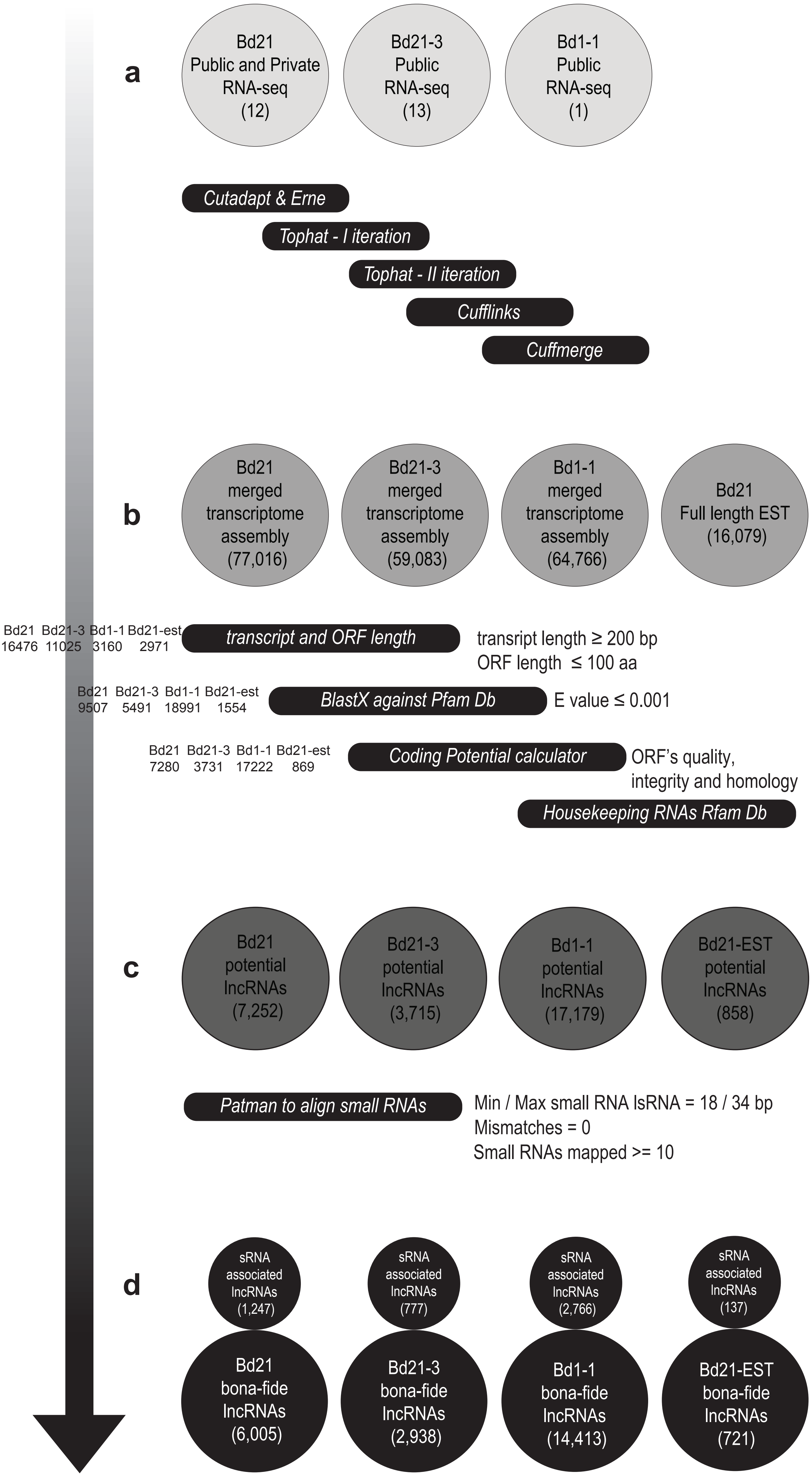

Supplemental Figure 2:

In silico validation of Bd21 lncRNAs annotation.

The figure shows three examples of sequence conservation between Bd21 lncRNAs and EST sequences. The output was generated with Dotter and mVista. (A) Dot plot comparisons using the software Dotter. Y-axis represents the lncRNA sequence and X-axis represents the EST. Diagonal ine indicates high sequence similarity between the Bd21 lncRNA and the Bd21 EST. (B) Sequence identity plot that compares Bd21 lncRNAs (top) against Bd21 EST (bottom) using the mVISTA pairwise alignment. The vertical y-axis indicates the percentage of identity to the reference (EST) ranging from 50% to 100%. The horizontal axis represents the coordinated base position between the two transcripts. The grey area indicates conserved noncoding regions.

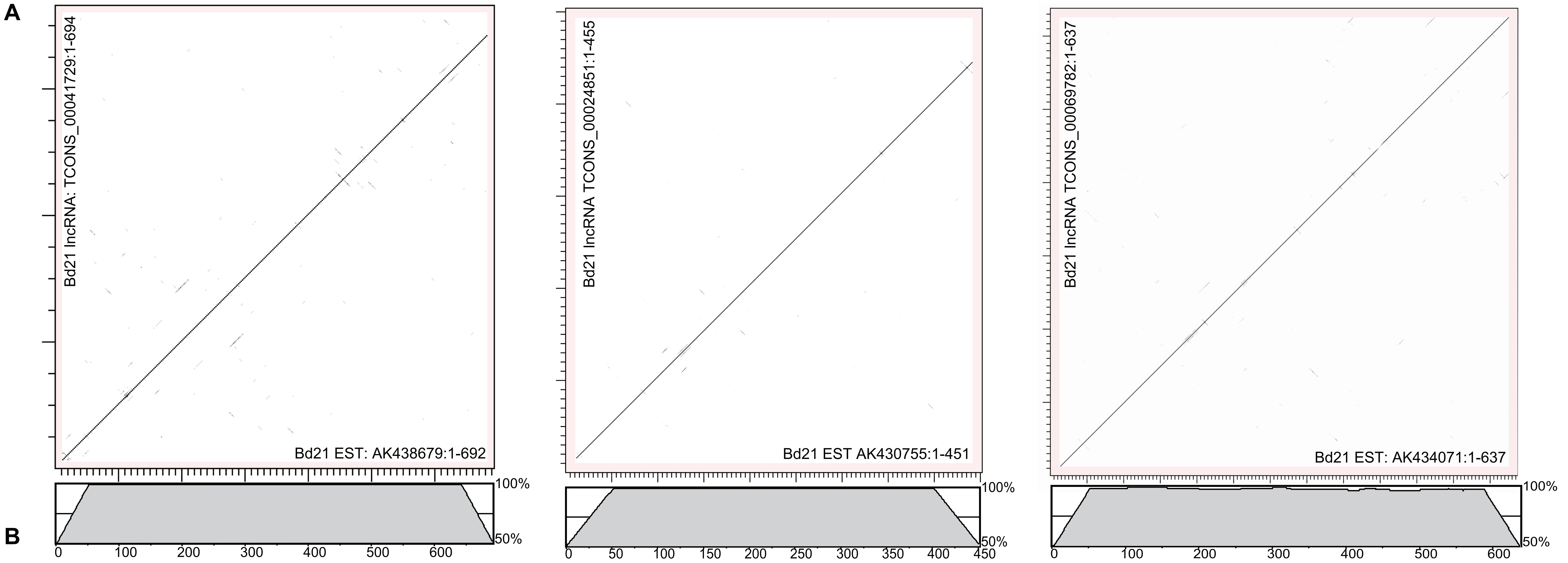

sequence1: AK438679:1-692
sequence2: TCONS_00041729:1-694(+)
X-axis:sequence1
Y-axis:% sequence identity

sequence1 AK430755:1-451
sequence2 TCONS_00024851:1-455 (+)
X-axis: sequence1
Y-axis:% sequence identity

sequence1 AK434071:1-637
sequence2 TCONS_00069782:1-637 (+)
X-axis: sequence1
Y-axis:% sequence identity

Supplemental Figure 3:
Description of microRNA genomic regions originated from lncRNAs genes. Representation of the genomic region of miR399a (A) and miR167c (B) showing that both miRNAs are originated from a lncRNA gene. LncRNAs gene structures are shown: black rectangles indicate exons and arrows indicates introns. The direction of arrows indicate a sense or antisense lncRNA gene. Numbers below the graph represent the chromosome coordinates. X axis represents genomic coordinates.

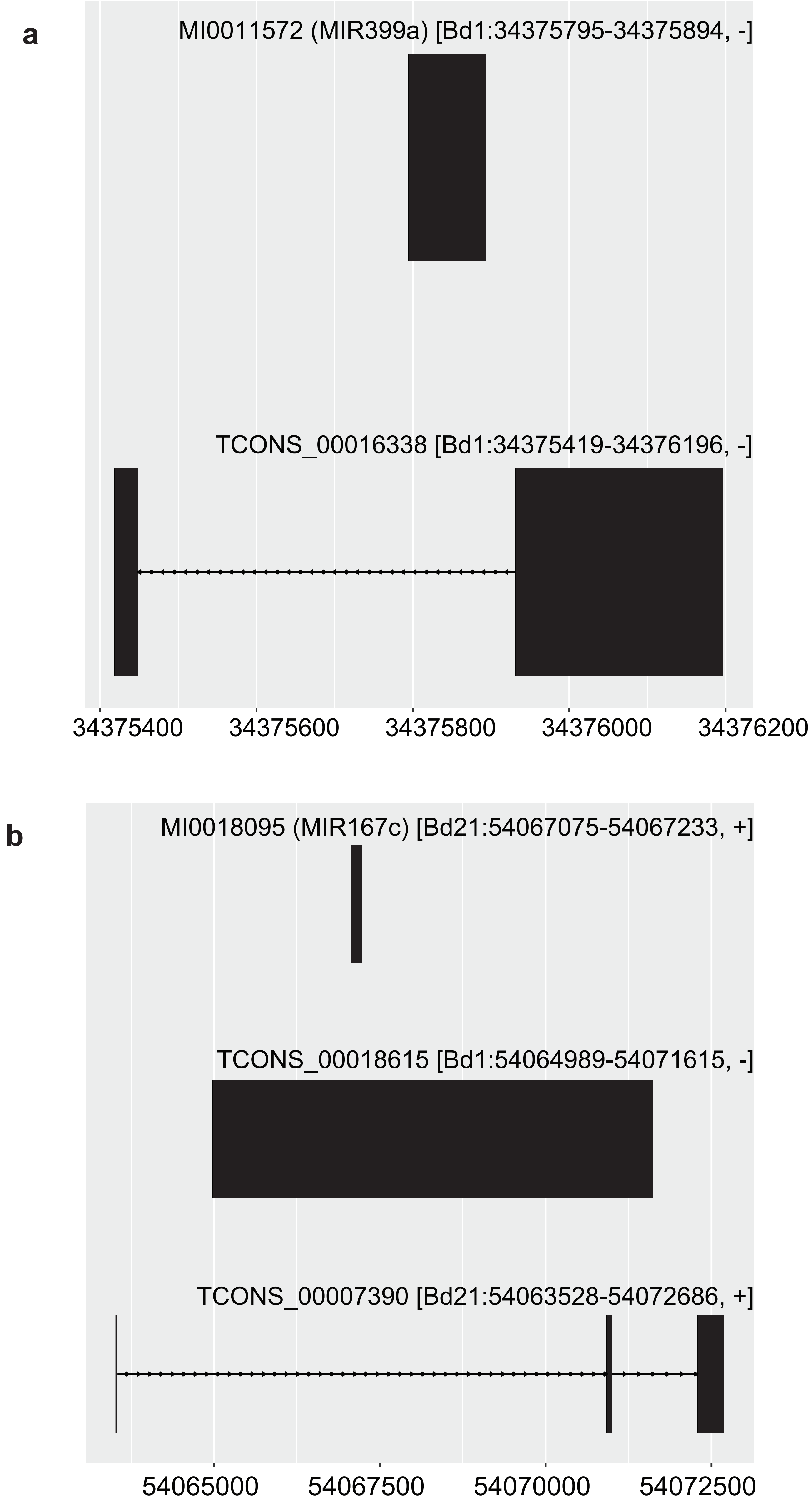

Supplemental Figure 4:
LncRNAs conservation across Bd lines.
The karyogram shows lncRNAs with a 90% sequence identity among Bd21, Bd21-3 and Bd1-1. Each horizontal black bar represents a Bd chromosome and vertical lines represent lncRNAs.
Homologous and syntenic lncRNAs are shown with the same color in all three Bd lines. X axis represents chromosome coordinates.

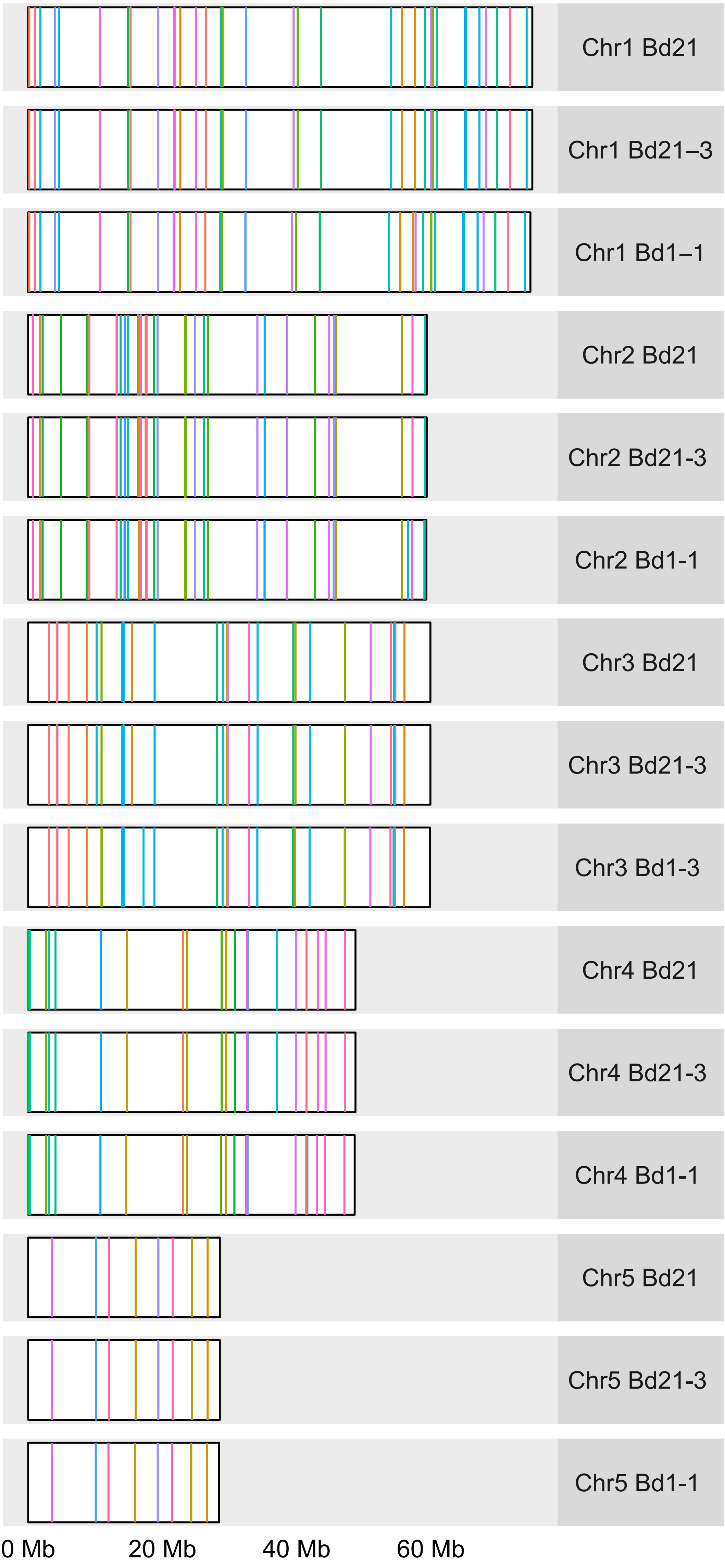

Supplemental Figure 5:
Bd21 and Bd21-3 specific lncRNAs.
(A) Pie Chart showing Bd21 specific lncRNAs. (B) Venn Diagram showing Bd21-3 specific lncRNAs considering the RNA-seq libraries produced by Mandadi et al. (2015). In both inbred lines we obtained specific lncRNA considering lncRNAs with RPKM>0 in each sample.

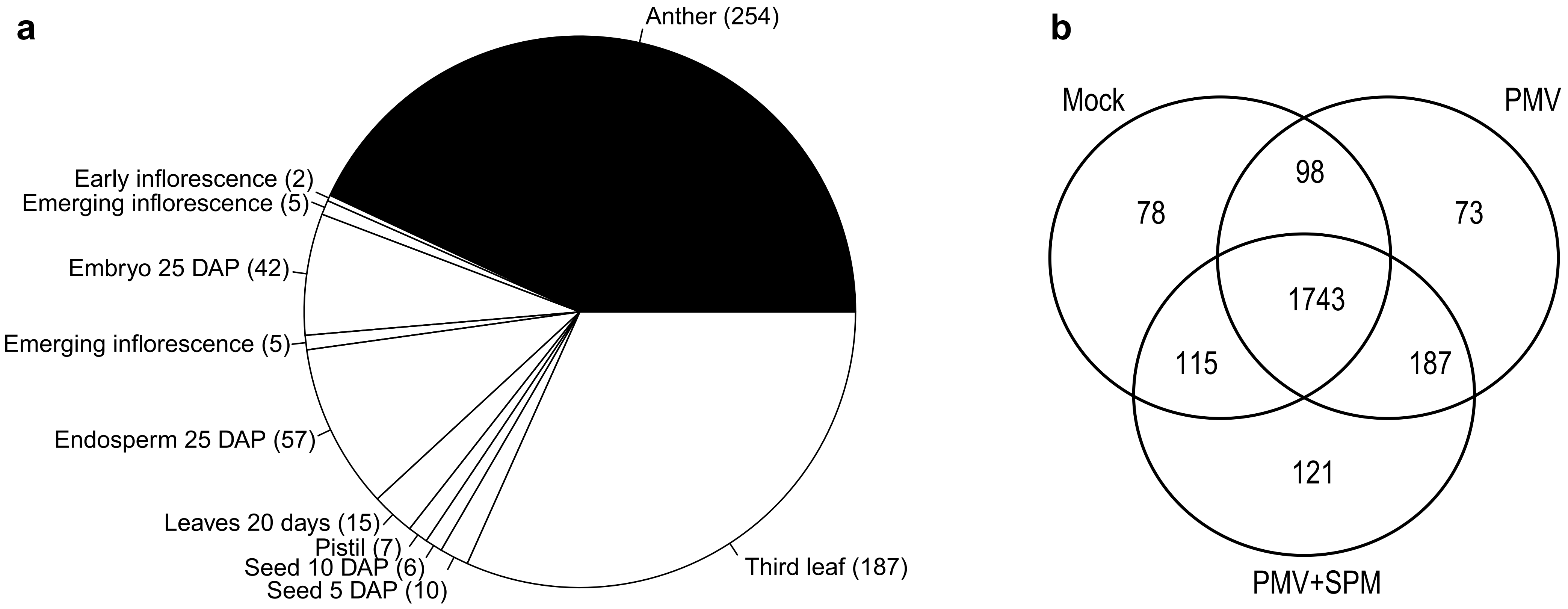

Supplemental Figure 6:
miR1122 targeting several Bd lncRNA transcripts.
Sequence alignments of Bd lncRNAs targeted by miR1122. Grey regions indicate sequence complementary of lncRNAs to the corresponding microRNA

	miR1122	3'	AGGUUUAUGCCUACAUAGAU	5'		
			:: ::::::::::::::::::			
Bd21	TCONS_00006529	5'	UCAAAAUACGGAUGUAUCUA	3'	score=1	range=161-180
Bd21	TCONS_00009915	5'	UCCAAAUAUGGAUGUAUCUA	3'	score=1	range=1213-1232
Bd21	TCONS_00016838	5'	UCCAAAUAUGGAUGUAUCUA	3'	score=1	range=110-129
Bd21	TCONS_00025302	5'	UCCAAAUAUGGAUGUAUCUA	3'	score=1	range=1394-1413
Bd21	TCONS_00035896	5'	UCCAAAUAUGGAUGUAUCUA	3'	score=1	range=51-70
Bd21	TCONS_00039769	5'	UCCAAAUAUGGAUGUAUCUA	3'	score=1	range=444-463
Bd21	TCONS_00049488	5'	CCCAAAUACGGAUGUAUCUA	3'	score=1	range=482-501
Bd21	TCONS_00012416	5'	UUCAAAUAUGGAUGUAUCUA	3'	score=1.5	range=434-453
Bd21	TCONS_00012417	5'	UUCAAAUAUGGAUGUAUCUA	3'	score=1.5	range=561-580
Bd21	TCONS_00012418	5'	UUCAAAUAUGGAUGUAUCUA	3'	score=1.5	range=506-525
Bd21	TCONS_00003489	5'	UCAAAAUAUGGAUGUAUCUA	3'	score=2	range=788-807
Bd21	TCONS_00023494	5'	CCCAAAUAUGGAUGUAUCUA	3'	score=2	range=1219-1238
Bd21	TCONS_00042010	5'	CCCAAAUAUGGAUGUAUCUA	3'	score=2	range=889-908
Bd21	TCONS_00042012	5'	CCCAAAUAUGGAUGUAUCUA	3'	score=2	range=1532-1551
Bd21	TCONS_00050077	5'	CCCAAAUAUGGAUGUAUCUA	3'	score=2	range=751-770
Bd21	TCONS_00068080	5'	CCCAAAUAUGGAUGUAUCUA	3'	score=2	range=853-872
Bd21-3	TCONS_00001338	5'	CCCAAAUACGGAUGUAUCUA	3'	score=1	range=426-445
Bd21-3	TCONS_00002741	5'	UCAAAAUAUGGAUGUAUCUA	3'	score=2	range=839-858
Bd21-3	TCONS_00027695	5'	CCCAAAUACGGAUGUAUCUA	3'	score=1	range=545-564
Bd21-3	TCONS_00032666	5'	CCCAAAUAUGGAUGUAUCUA	3'	score=2	range=1532-1551
Bd21-3	TCONS_00038427	5'	CCCAAAUACGGAUGUAUCUA	3'	score=1	range=482-501
Bd21-3	TCONS_00038899	5'	CCCAAAUAUGGAUGUAUCUA	3'	score=2	range=751-770
Bd21-3	TCONS_00043006	5'	UCCAAAUAUGGAUGUAUCUA	3'	score=1	range=514-533
Bd21-3	TCONS_00052696	5'	UCCAAAUAUGGAUGUAUCUA	3'	score=1	range=178-197
Bd1-1	TCONS_10828.1	5'	CCCAAAUACGGAUGUAUCUA	3'	score=1	range=736-755
Bd1-1	TCONS_10915.1	5'	CCCAAAUAUGGAUGUAUCUA	3'	score=2	range=198-217
Bd1-1	TCONS_10915.1	5'	CCCAAAUAUGGAUGUAUCUA	3'	score=2	range=2212-2231
Bd1-1	TCONS_12293.1	5'	CCCAAAUAUGGAUGUAUCUA	3'	score=2	range=238-257
Bd1-1	TCONS_15684.1	5'	CCCAAAUACGGAUGUAUUUA	3'	score=2	range=350-369
Bd1-1	TCONS_16720.1	5'	CCCAAAUAUGGAUGUAUCUA	3'	score=2	range=573-592
Bd1-1	TCONS_17230.1	5'	CCCAAAUAUGGAUGUAUCUA	3'	score=2	range=1235-1254
Bd1-1	TCONS_21004.1	5'	CCCAAAUAUGGAUGUAUCUA	3'	score=2	range=911-930
Bd1-1	TCONS_22078.1	5'	CCCAAAUAUGGAUGUAUCUA	3'	score=2	range=846-865
Bd1-1	TCONS_22384.1	5'	UCCAGGUAUGGAUGUAUCUA	3'	score=2	range=1111-1130
Bd1-1	TCONS_23435.1	5'	CCCAAAUACGGAUGUAUCUA	3'	score=1	range=325-344
Bd1-1	TCONS_24093.1	5'	CCCAAAUAUGGAUGUAUCUA	3'	score=2	range=480-499
Bd1-1	TCONS_25207.1	5'	UCCAAAUAUGGAUGUAUCUA	3'	score=1	range=354-373
Bd1-1	TCONS_25435.1	5'	CCCAAAUAUGGAUGUAUCUA	3'	score=2	range=1762-1781
Bd1-1	TCONS_26050.1	5'	UCCAAAUAUGGAUGUAUCUG	3'	score=1.5	range=234-253
Bd1-1	TCONS_26580.1	5'	CCCAAAUAUGGAUGUAUCUA	3'	score=2	range=618-637
Bd1-1	TCONS_26637.1	5'	CCCAAAUAUGGAUGUAUCUA	3'	score=2	range=91-110
Bd1-1	TCONS_29166.1	5'	UCCAAAAAUGGAUGUAUCUA	3'	score=2	range=1468-1487
Bd1-1	TCONS_31935.1	5'	UCCAAAUGUGGAUGUAUCUA	3'	score=2	range=29-48
Bd1-1	TCONS_32240.1	5'	CCCAAAUAUGGAUGUAUCUA	3'	score=2	range=5348-5367
Bd1-1	TCONS_32403.1	5'	CCCAAAUAUGGAUGUAUCUA	3'	score=2	range=333-352
Bd1-1	TCONS_3412.1	5'	UCCAAAUAUGGAUGUAUCUA	3'	score=1	range=902-921
Bd1-1	TCONS_34246.1	5'	CCCAAAUAUGGAUGUAUCUA	3'	score=2	range=5-24
Bd1-1	TCONS_34901.1	5'	CCCAAAUAUGGAUGUAUCUA	3'	score=2	range=1538-1557
Bd1-1	TCONS_35470.1	5'	UCCAAAUAUGGAUGUAUUUA	3'	score=2	range=1-20
Bd1-1	TCONS_41865.1	5'	CCCAAAUAUGGAUGUAUCUA	3'	score=2	range=1527-1546
Bd1-1	TCONS_42310.1	5'	CCCAAAUACGGAUGUAUCUA	3'	score=1	range=527-546
Bd1-1	TCONS_43365.1	5'	UCAAAAUAUGGAUGUAUCUA	3'	score=2	range=76-95
Bd1-1	TCONS_43436.1	5'	CCCAAAUAUGGAUGUAUCUA	3'	score=2	range=716-735
Bd1-1	TCONS_43499.1	5'	UCUAAAUAUGGAUGUAUCUA	3'	score=1.5	range=108-127
Bd1-1	TCONS_44372.1	5'	CCCAAAUAUGGAUGUAUCUA	3'	score=2	range=3586-3605
Bd1-1	TCONS_44530.1	5'	CCCAAAUAUGGAUGUAUCUA	3'	score=2	range=1238-1257
Bd1-1	TCONS_46454.1	5'	CCCAAAUAUGGAUGUAUCUA	3'	score=2	range=407-426
Bd1-1	TCONS_48497.1	5'	CCCAAAUAUGGAUGUAUCUA	3'	score=2	range=827-846
Bd1-1	TCONS_50071.1	5'	CCCAAAUAUGGAUGUAUCUA	3'	score=2	range=122-141
Bd1-1	TCONS_52955.1	5'	CCCAAAUAUGGAUGUAUCUA	3'	score=2	range=853-872
Bd1-1	TCONS_53893.1	5'	CCCAAAUAUGGAUGUAUCUA	3'	score=2	range=1781-1800
Bd1-1	TCONS_57242.1	5'	ACCAAAUAUGGAUGUAUCUA	3'	score=2	range=5-24
Bd1-1	TCONS_57847.1	5'	UCCAAAUAUGGAUGUAUCUA	3'	score=1	range=425-444
Bd1-1	TCONS_59827.1	5'	UCUAGAUACGGAUGUAUCUA	3'	score=1	range=684-703
Bd1-1	TCONS_6107.1	5'	GCCAAAUACGGAUGUAUCUA	3'	score=1	range=16-35
Bd1-1	TCONS_9323.1	5'	CCCAAAUAUGGAUGUAUCUA	3'	score=2	range=491-510
Bd1-1	TCONS_9323.2	5'	CCCAAAUAUGGAUGUAUCUA	3'	score=2	range=592-611
Bd1-1	TCONS_9424.1	5'	CCCAAAUAUGGAUGUAUCUA	3'	score=2	range=726-745

Supplemental Figure 7:
miR5174 targeting several Bd lncRNA transcripts.
Sequence alignments of Bd lncRNAs targeted by miR5174. Grey regions indicate sequence complementary of lncRNAs to the corresponding microRNAs.

```
                 miR5174e-5p.2                3'       GAAAUACCUUGUCUCCCUCAU 5'
                 miR5174e-5p.1                3'  GGUUAGAAAUACCUUGUCUCC      5'
                 miR5174b-5p                  3'  GGUUAGAAAUACCUUGUCUCC      5'
                 miR5174f-5p                  3'  GGUUGGAAAUACUUUGCCUCC      5'
                 miR5174a-5p                  3' CGGUUAGAAAUACCUUGCCUC       5'
                 miR5174c-5p                  3' CGGUUAGAAGUAUCUUGCCUCCCU    5'
                 miR5174d-5p                  3' CGGUUAGAAAUACUUUGCCUCCCU    5'
                                                 ::::::::::::..:::::::::
Bd21      TCONS_00030507:miR5174d-5p         5' GCCAAUCUUUAUGGGACGGAGGGA    3'    score=2      range=559-582
Bd21      TCONS_00030508:miR5174d-5p         5' GCCAAUCUUUAUGGGACGGAGGGA    3'    score=2      range=559-582
Bd21      TCONS_00030509:miR5174d-5p         5' GCCAAUCUUUAUGGGACGGAGGGA    3'    score=2      range=559-582
Bd21      TCONS_00042655:miR5174d-5p         5' GCCAACCUUUAUGAAACGGAGGGA    3'    score=1      range=926-949
Bd21-3    TCONS_00023796:miR5174d-5p         5' GCCAAUCUUUAUGGGACGGAGGGA    3'    score=2      range=575-598
Bd21-3    TCONS_00023797:miR5174d-5p         5' GCCAAUCUUUAUGGGACGGAGGGA    3'    score=2      range=559-582
Bd1-1     TCONS_13517.1:miR5174d-5p          5' GCCAAUCUUUAUGAAAUGGAGGGA    3'    score=1      range=395-418
Bd1-1     TCONS_20978.1:miR5174d-5p          5' GCCAAUCUUUAUGAAACGGAAGGA    3'    score=2      range=1675-1698
Bd1-1     TCONS_45809.1:miR5174d-5p          5' GCCAAUCUUUGUGAAAUGGAGGGA    3'    score=1.5    range=409-432
Bd1-1     TCONS_53823.1:miR5174d-5p          5' GCCAAUUUUUAUGGAACGGAGGGA    3'    score=1.5    range=508-531
Bd1-1     TCONS_58904.1:miR5174d-5p          5' GCCAAUCUUUAUGAAACGGAAGGA    3'    score=2      range=1025-1048
Bd1-1     TCONS_61903.1:miR5174d-5p          5' GUCAAUCUUUAUGGAACGGAGGGA    3'    score=1.5    range=303-326
Bd1-1     TCONS_61952.1:miR5174d-5p          5' GCCAAUCUUUAUGAAACGGAGGGA    3'    score=0      range=553-576
Bd1-1     TCONS_6930.1:miR5174d-5p           5' GCCAACCUUUAUGAAACGGAGGGA    3'    score=1      range=8-31
Bd1-1     TCONS_53823.1:miR5174c-5p          5' GCCAAUUUUUAUGGAACGGAGGGA    3'    score=2      range=508-531
Bd1-1     TCONS_61903.1:miR5174c-5p          5' GUCAAUCUUUAUGGAACGGAGGGA    3'    score=2      range=303-326
Bd21      TCONS_00030507:miR5174a-5p         5' GCCAAUCUUUAUGGGACGGAG       3'    score=1      range=559-579
Bd21      TCONS_00030508:miR5174a-5p         5' GCCAAUCUUUAUGGGACGGAG       3'    score=1      range=559-579
Bd21      TCONS_00030509:miR5174a-5p         5' GCCAAUCUUUAUGGGACGGAG       3'    score=1      range=559-579
Bd21      TCONS_00031306:miR5174a-5p         5' GCCAAUAUUUAUGGGACGGAG       3'    score=2      range=119-139
Bd21-3    TCONS_00023796:miR5174a-5p         5' GCCAAUCUUUAUGGGACGGAG       3'    score=1      range=575-595
Bd21-3    TCONS_00023797:miR5174a-5p         5' GCCAAUCUUUAUGGGACGGAG       3'    score=1      range=559-579
Bd21-3    TCONS_00025119:miR5174a-5p         5' GCCAAUCUUUAUGGAACAGAG       3'    score=2      range=378-398
Bd1-1     TCONS_16870.1:miR5174a-5p          5' CCCAACCUUUAUGGAACGGAG       3'    score=2        range=13-33
Bd1-1     TCONS_40876.1:miR5174a-5p          5' GCCGAUUUUUAUGGAACGGAG       3'    score=1      range=369-389
Bd1-1     TCONS_53823.1:miR5174a-5p          5' GCCAAUUUUUAUGGAACGGAG       3'    score=0.5    range=508-528
Bd1-1     TCONS_61903.1:miR5174a-5p          5' GUCAAUCUUUAUGGAACGGAG       3'    score=0.5    range=303-323
Bd1-1     TCONS_61952.1:miR5174a-5p          5' GCCAAUCUUUAUGAAACGGAG       3'    score=2      range=553-573
Bd21      TCONS_00025822:miR5174f-5p         5'  CCAACCUUUAUGAAAUGGAGG      3'    score=1      range=1391-1411
Bd21      TCONS_00042655:miR5174f-5p         5'  CCAACCUUUAUGAAACGGAGG      3'    score=0      range=927-947
Bd21      TCONS_00060336:miR5174f-5p         5'  CCAACCUUUGUGAAAUGGAGG      3'    score=2      range=524-544
Bd1-1     TCONS_12509.1:miR5174f-5p          5'  CCAACCUUUGUGAAAUGGAGG      3'    score=2      range=847-867
Bd1-1     TCONS_13517.1:miR5174f-5p          5'  CCAAUCUUUAUGAAAUGGAGG      3'    score=1.5    range=396-416
Bd1-1     TCONS_16870.1:miR5174f-5p          5'  CCAACCUUUAUGGAACGGAGG      3'    score=1      range=14-34
Bd1-1     TCONS_24920.1:miR5174f-5p          5'  CCAACCUUUAUGAAACCGAGG      3'    score=2      range=62-82
Bd1-1     TCONS_4852.1:miR5174f-5p           5'  CCAACCUUUGUGAAAUGGAGG      3'    score=2      range=839-859
Bd1-1     TCONS_53653.1:miR5174f-5p          5'  CCAACCUUUGUGAAAUGGAGG      3'    score=2      range=1333-1353
Bd1-1     TCONS_53823.1:miR5174f-5p          5'  CCAAUUUUUAUGGAACGGAGG      3'    score=2      range=509-529
Bd1-1     TCONS_61903.1:miR5174f-5p          5'  UCAAUCUUUAUGGAACGGAGG      3'    score=2      range=304-324
Bd1-1     TCONS_61952.1:miR5174f-5p          5'  CCAAUCUUUAUGAAACGGAGG      3'    score=0.5    range=554-574
Bd1-1     TCONS_6930.1:miR5174f-5p           5'  CCAACCUUUAUGAAACGGAGG      3'    score=0      range=9-29
Bd21      TCONS_00030507:miR5174b-5p         5'  CCAAUCUUUAUGGGACGGAGG      3'    score=2      range=560-580
Bd21      TCONS_00030508:miR5174b-5p         5'  CCAAUCUUUAUGGGACGGAGG      3'    score=2      range=560-580
Bd21      TCONS_00030509:miR5174b-5p         5'  CCAAUCUUUAUGGGACGGAGG      3'    score=2      range=560-580
Bd21-3    TCONS_00023796:miR5174b-5p         5'  CCAAUCUUUAUGGGACGGAGG      3'    score=2      range=576-596
Bd21-3    TCONS_00023797:miR5174b-5p         5'  CCAAUCUUUAUGGGACGGAGG      3'    score=2      range=560-580
Bd21-3    TCONS_00025119:miR5174b-5p         5'  CCAAUCUUUAUGGAACAGAGG      3'    score=0      range=379-399
Bd1-1     TCONS_16870.1:miR5174b-5p          5'  CCAACCUUUAUGGAACGGAGG      3'    score=2      range=14-34
Bd1-1     TCONS_40876.1:miR5174b-5p          5'  CCGAUUUUUAUGGAACGGAGG      3'    score=2      range=370-390
Bd1-1     TCONS_53823.1:miR5174b-5p          5'  CCAAUUUUUAUGGAACGGAGG      3'    score=1.5    range=509-529
Bd1-1     TCONS_61903.1:miR5174b-5p          5'  UCAAUCUUUAUGGAACGGAGG      3'    score=1.5    range=304-324
Bd21      TCONS_00030507:miR5174e-5p.1       5'  CCAAUCUUUAUGGGACGGAGG      3'    score=2      range=560-580
Bd21      TCONS_00030508:miR5174e-5p.1       5'  CCAAUCUUUAUGGGACGGAGG      3'    score=2      range=560-580
Bd21      TCONS_00030509:miR5174e-5p.1       5'  CCAAUCUUUAUGGGACGGAGG      3'    score=2      range=560-580
Bd21-3    TCONS_00023796:miR5174e-5p.1       5'  CCAAUCUUUAUGGGACGGAGG      3'    score=2      range=576-596
Bd21-3    TCONS_00023797:miR5174e-5p.1       5'  CCAAUCUUUAUGGGACGGAGG      3'    score=2      range=560-580
Bd21-3    TCONS_00025119:miR5174e-5p.1       5'  CCAAUCUUUAUGGAACAGAGG      3'    score=0      range=379-399
Bd1-1     TCONS_16870.1:miR5174e-5p.1        5'  CCAACCUUUAUGGAACGGAGG      3'    score=2      range=14-34
Bd1-1     TCONS_40876.1:miR5174e-5p.1        5'  CCGAUUUUUAUGGAACGGAGG      3'    score=2      range=370-390
Bd1-1     TCONS_53823.1:miR5174e-5p.1        5'  CCAAUUUUUAUGGAACGGAGG      3'    score=1.5    range=509-529
Bd1-1     TCONS_61903.1:miR5174e-5p.1        5'  UCAAUCUUUAUGGAACGGAGG      3'    score=1.5    range=304-324
Bd21      TCONS_00030507:miR5174e-5p.2       5'       CUUUAUGGGACGGAGGGAGUA 3'    score=2      range=565-585
Bd21      TCONS_00030508:miR5174e-5p.2       5'       CUUUAUGGGACGGAGGGAGUA 3'    score=2      range=565-585
Bd21      TCONS_00030509:miR5174e-5p.2       5'       CUUUAUGGGACGGAGGGAGUA 3'    score=2      range=565-585
Bd21      TCONS_00042655:miR5174e-5p.2       5'       CUUUAUGAAACGGAGGGAGUA 3'    score=2      range=932-952
Bd21-3    TCONS_00023796:miR5174e-5p.2       5'       CUUUAUGGGACGGAGGGAGUA 3'    score=2      range=581-601
Bd21-3    TCONS_00023797:miR5174e-5p.2       5'       CUUUAUGGGACGGAGGGAGUA 3'    score=2      range=565-585
Bd21-3    TCONS_00025119:miR5174e-5p.2       5'       CUUUAUGGAACAGAGGGAGUA 3'    score=0      range=384-404
Bd1-1     TCONS_10611.1:miR5174e-5p.2        5'       AUUUA-GGAACAGAGGGAGUA 3'    score=2      range=943-962
Bd1-1     TCONS_11462.1:miR5174e-5p.2        5'       AAUUAUGGAACAGAGGGAGUA 3'    score=2      range=62-82
Bd1-1     TCONS_16870.1:miR5174e-5p.2        5'       CUUUAUGGAACGGAGGGAGUA 3'    score=1      range=19-39
Bd1-1     TCONS_31107.1:miR5174e-5p.2        5'       AUUUA-GGAACAGAGGGAGUA 3'    score=2      range=391-410
Bd1-1     TCONS_3723.1:miR5174e-5p.2         5'       AUUUA-GGAACAGAGGGAGUA 3'    score=2      range=620-639
Bd1-1     TCONS_43697.1:miR5174e-5p.2        5'       UAUUAUGGAACAGAGGGAGUA 3'    score=1.5    range=795-815
Bd1-1     TCONS_44478.1:miR5174e-5p.2        5'       AUUUA-GGAACAGAGGGAGUA 3'    score=2      range=207-226
Bd1-1     TCONS_53823.1:miR5174e-5p.2        5'       UUUUAUGGAACGGAGGGAGUA 3'    score=1.5    range=514-534
Bd1-1     TCONS_55385.1:miR5174e-5p.2        5'       AUUUA-GGAACAGAGGGAGUA 3'    score=2      range=383-402
Bd1-1     TCONS_57026.1:miR5174e-5p.2        5'       CUUUAAGGAACGGAGGGAGUA 3'    score=2      range=914-934
Bd1-1     TCONS_58032.1:miR5174e-5p.2        5'       AAUUAUGGAACAGAGGGAGUA 3'    score=2      range=17-37
Bd1-1     TCONS_59889.1:miR5174e-5p.2        5'       AAUUAUGGAACAGAGGGAGUA 3'    score=2      range=352-372
Bd1-1     TCONS_61903.1:miR5174e-5p.2        5'       CUUUAUGGAACGGAGGGAGUA 3'    score=1      range=309-329
Bd1-1     TCONS_61952.1:miR5174e-5p.2        5'       CUUUAUGAAACGGAGGGAGUA 3'    score=2      range=559-579
Bd1-1     TCONS_6930.1:miR5174e-5p.2         5'       CUUUAUGAAACGGAGGGAGUA 3'    score=2      range=14-34
Bd1-1     TCONS_7322.1:miR5174e-5p.2         5'       AUUUA-GGAACAGAGGGAGUA 3'    score=2      range=182-201
```

```
                 miR5174b-3p                   3'      GGGAGGCAAGGUAUUUCCAAC 5'
                 miR5174e-3p.1                 3'      GGGAGGCAAGGUAUUUCCAAC 5'
                 miR5174d-3p                   3'   UGAGAGAGGCAAGGUAUUUCUAAC 5'
                 miR5174e-3p.2                 3' GAUGAGGGAGGCAAGGUAUUU      5'
                                                   :::::::::::::::::::
Bd21      TCONS_00009395:miR5174e-3p.2         5' AUACUCCCUCCGUUCCAUAAU      3'    score=2      range=1085-1105
Bd21      TCONS_00014862:miR5174e-3p.2          5' CUACUCCCUCCGUUCC-UAAA      3'    score=2      range=147-166
Bd21      TCONS_00015543:miR5174e-3p.2         5' CUACUCCCUCCGUUCC-UAAA      3'    score=2      range=559-578
Bd21      TCONS_00016206:miR5174e-3p.2         5' CUACUCCCUCCGUUCCAUAAU      3'    score=1      range=559-579
Bd21      TCONS_00018213:miR5174e-3p.2         5' CUACUCCCUCUGUUCCAUAAU      3'    score=2      range=716-736
Bd21      TCONS_00031306:miR5174e-3p.2         5' GUAGUCCCUCCGUUCCAUAAA      3'    score=2      range=59-79
Bd21      TCONS_00038198:miR5174e-3p.2         5' GUACUCCCUCCGUUCCAUAAU      3'    score=2      range=1093-1113
Bd21      TCONS_00042655:miR5174e-3p.2         5' CUACUCCCUCCGUUCCAUAAA      3'    score=0      range=866-886
Bd21      TCONS_00060336:miR5174e-3p.2         5' AUACUCCCUCCGUUCCAUAAA      3'    score=1      range=1156-1176
Bd21      TCONS_00070470:miR5174e-3p.2         5' CUACUCCCUCCGUCCCAUAAA      3'    score=2      range=1883-1903
Bd21-3    TCONS_00005858:miR5174e-3p.2         5' AUACUCCCUCCGUUCCAUAAU      3'    score=2      range=1115-1135
Bd21-3    TCONS_00020984:miR5174e-3p.2         5' GUACUCCCUCCGUUCCAUAAU      3'    score=2      range=142-162
Bd21-3    TCONS_00022657:miR5174e-3p.2         5' CUACUCCCUCCGUUCCAUAAU      3'    score=1      range=577-597
Bd21-3    TCONS_00025119:miR5174e-3p.2         5' CUGCUCCCUCCGUUUCAUAAA      3'    score=1.5    range=318-338
Bd21-3    TCONS_00027215:miR5174e-3p.2         5' CUACUCCCUCCGUUCC-UAAA      3'    score=2      range=849-868
Bd21-3    TCONS_00029781:miR5174e-3p.2         5' GUACUCCCUCCGUUCCAUAAU      3'    score=2      range=1125-1145
Bd21-3    TCONS_00036023:miR5174e-3p.2         5' CUACUCCCUCCGAUCCAUAAA      3'    score=2      range=832-852
Bd21-3    TCONS_00038651:miR5174e-3p.2         5' CUACUCCCUCCGUUCCAUAAU      3'    score=1      range=407-427
Bd21-3    TCONS_00048434:miR5174e-3p.2         5' CUACUCCCUCCGUUCCUUAAA      3'    score=2      range=656-676
Bd21-3    TCONS_00049063:miR5174e-3p.2         5' AUACUCCCUCCGUUCCAUAAU      3'    score=2      range=286-306
Bd21-3    TCONS_00051345:miR5174e-3p.2         5' UUACUCCCUCCGUUCCAUAAU      3'    score=1.5    range=494-514
Bd1-1     TCONS_10675.1:miR5174e-3p.2          5' AUACUCCCUCCGUUCCAUAAU      3'    score=2      range=557-577
Bd1-1     TCONS_11205.1:miR5174e-3p.2          5' CUACUCCCUCCGUUCC-UAAA      3'    score=2      range=2417-2436
Bd1-1     TCONS_12509.1:miR5174e-3p.2          5' CUACUCCCUCUGUUCCAUAAU      3'    score=2      range=2398-2418
Bd1-1     TCONS_14774.1:miR5174e-3p.2          5' GUACUCCCUCCGUUCCAUAAU      3'    score=2      range=441-461
Bd1-1     TCONS_15085.1:miR5174e-3p.2          5' CUACUCCCUCCGUUCCAUAAU      3'    score=1      range=417-437
Bd1-1     TCONS_15684.1:miR5174e-3p.2          5' AUACUCCCUCCGUUCCAUAAU      3'    score=2      range=313-333
Bd1-1     TCONS_17331.1:miR5174e-3p.2          5' CUACUCCCUCCGUUCC-UAAA      3'    score=2      range=88-107
Bd1-1     TCONS_20978.1:miR5174e-3p.2          5' AUACUCCCUCUGUUCCAUAAA      3'    score=2      range=1615-1635
Bd1-1     TCONS_23381.1:miR5174e-3p.2          5' CUACUCCCUCCGUUCCAUAAU      3'    score=1      range=153-173
Bd1-1     TCONS_23382.1:miR5174e-3p.2          5' UUACUCCCUCCGUUCCAUAAU      3'    score=1.5    range=320-340
Bd1-1     TCONS_25304.1:miR5174e-3p.2          5' CUACUACCUCCGUUCCAUAAU      3'    score=2      range=261-281
Bd1-1     TCONS_2609.1:miR5174e-3p.2           5' UUACUCUCUCCGUUCCAUAAU      3'    score=2      range=532-552
Bd1-1     TCONS_26523.1:miR5174e-3p.2          5' GUACUCCCUCCGUUCCAUAAU      3'    score=2      range=603-623
Bd1-1     TCONS_28498.1:miR5174e-3p.2          5' CUGCUCCCUCCGUUCCAUAAU      3'    score=1.5    range=464-484
Bd1-1     TCONS_28801.1:miR5174e-3p.2          5' CUACUCCCUCCGUUCC-UAAA      3'    score=2      range=615-634
Bd1-1     TCONS_31521.1:miR5174e-3p.2          5' GUACUCCCUCCGUUCCAUAAU      3'    score=2      range=1124-1144
Bd1-1     TCONS_35488.1:miR5174e-3p.2          5' GUACUCCCUCCGUUCCAUAAU      3'    score=2      range=4019-4039
Bd1-1     TCONS_35617.1:miR5174e-3p.2          5' CUACUCCCUCCGUUCCAUAAA      3'    score=0      range=659-679
Bd1-1     TCONS_35635.1:miR5174e-3p.2          5' AUACUCCCUCCGUUCCAUAAU      3'    score=2      range=444-464
Bd1-1     TCONS_37188.2:miR5174e-3p.2          5' GUACUCCCUCCGUUCCAUAAU      3'    score=2      range=1554-1574
Bd1-1     TCONS_3723.1:miR5174e-3p.2           5' CUACUCCCUCCGUUCC-UAAA      3'    score=2      range=554-573
Bd1-1     TCONS_37817.1:miR5174e-3p.2          5' CUACUCCCUCCGUUCCAUAAU      3'    score=1      range=168-188
Bd1-1     TCONS_41572.1:miR5174e-3p.2          5' CUACUCCCUCCGUUCCAUAAU      3'    score=1      range=236-256
Bd1-1     TCONS_43023.1:miR5174e-3p.2          5' CUACUCCCUCCGUUCCAUAAU      3'    score=1      range=174-194
Bd1-1     TCONS_43480.1:miR5174e-3p.2          5' GUACUCCCUCCGUUCCAUAAU      3'    score=2      range=2494-2514
Bd1-1     TCONS_43602.1:miR5174e-3p.2          5' CUACUCCCUCCGUUUCAUAAG      3'    score=1.5    range=6-26
Bd1-1     TCONS_44372.1:miR5174e-3p.2          5' GUACUCCCUCCGUUCCAUAAU      3'    score=2      range=3549-3569
Bd1-1     TCONS_46605.1:miR5174e-3p.2          5' CUACUCCCUCCAUUCCAUAAA      3'    score=2      range=445-465
Bd1-1     TCONS_49774.1:miR5174e-3p.2          5' AUACUCCCUCCGUUCCAUAAU      3'    score=2      range=686-706
Bd1-1     TCONS_51893.1:miR5174e-3p.2          5' GUACUCCCUCCGUUCCAUAAU      3'    score=2      range=1499-1519
Bd1-1     TCONS_53823.1:miR5174e-3p.2          5' GUACUCCCUCCGUUCCGUAAA      3'    score=2      range=448-468
Bd1-1     TCONS_54082.1:miR5174e-3p.2          5' UUACUCCCUCCGUUCCAUAAU      3'    score=1.5    range=369-389
Bd1-1     TCONS_54454.1:miR5174e-3p.2          5' CUACUCCCUCCGUUUCAUAAU      3'    score=2      range=349-369
Bd1-1     TCONS_54611.1:miR5174e-3p.2          5' CUACUCCCUCCGUUUCAUAAU      3'    score=2      range=393-413
Bd1-1     TCONS_56386.1:miR5174e-3p.2          5' CUACUCCCUCUGUUCCAUAAU      3'    score=2      range=639-659
Bd1-1     TCONS_56386.1:miR5174e-3p.2          5' GUACUCCCUCCGUUCCAUAAU      3'    score=2      range=766-786
Bd1-1     TCONS_56688.1:miR5174e-3p.2          5' CUACUCCCUCCGAUCCAUAAA      3'    score=2      range=983-1003
Bd1-1     TCONS_58904.1:miR5174e-3p.2          5' CUACUCCCUCCAUUCCAUAAA      3'    score=2      range=965-985
Bd1-1     TCONS_59492.1:miR5174e-3p.2          5' CUACUCCCUCCGUCCCAUAAA      3'    score=2      range=2004-2024
Bd1-1     TCONS_60276.1:miR5174e-3p.2          5' AUACUCCCUCCGUUCCAUAAU      3'    score=2      range=1351-1371
Bd1-1     TCONS_8737.1:miR5174e-3p.2           5' GUACUCCCUCCGUUCCAUAAU      3'    score=2      range=533-553
Bd21      TCONS_00031306:miR5174d-3p           5'   AGUCCCUCCGUUCCAUAAAGAUUG 3'    score=2      range=61-84
Bd21      TCONS_00042655:miR5174d-3p           5'   ACUCCCUCCGUUCCAUAAAGAUUG 3'    score=1      range=868-891
Bd21      TCONS_00060336:miR5174d-3p           5'   ACUCCCUCCGUUCCAUAAAGAUUG 3'    score=1      range=1158-1181
Bd1-1     TCONS_19882.1:miR5174d-3p            5'   ACUCUCUCUGUUUCAUAAAGAUUG 3'    score=1.5    range=524-547
Bd1-1     TCONS_20978.1:miR5174d-3p            5'   ACUCCCUCUGUUCCAUAAAGAUUG 3'    score=1.5    range=1617-1640
Bd1-1     TCONS_53823.1:miR5174d-3p            5'   ACUCCCUCCGUUCCGUAAAGAUUG 3'    score=2      range=450-473
Bd1-1     TCONS_61903.1:miR5174d-3p            5'   ACUC-CUCCGUUUCAUAAAGAUUG 3'    score=2      range=246-268
Bd21      TCONS_00031306:miR5174e-3p.1         5'      CCCUCCGUUCCAUAAAGAUUG 3'    score=2      range=64-84
Bd21      TCONS_00042655:miR5174e-3p.1         5'      CCCUCCGUUCCAUAAAGAUUG 3'    score=2      range=871-891
Bd21      TCONS_00060336:miR5174e-3p.1         5'      CCCUCCGUUCCAUAAAGAUUG 3'    score=2      range=1161-1181
Bd21      TCONS_00031306:miR5174b-3p           5'      CCCUCCGUUCCAUAAAGAUUG 3'    score=2      range=64-84
Bd21      TCONS_00042655:miR5174b-3p           5'      CCCUCCGUUCCAUAAAGAUUG 3'    score=2      range=871-891
Bd21      TCONS_00060336:miR5174b-3p           5'      CCCUCCGUUCCAUAAAGAUUG 3'    score=2      range=1161-1181
```

Supplemental Figure 8:
miR5181 targeting several Bd lncRNA transcripts.
Sequence alignments of Bd lncRNAs targeted by miR5181. Grey regions indicate sequence complementary of lncRNAs to the corresponding microRNAs.

a

```
Bd1-1  TCONS_10960.1:miR5181b-5p       5' UGACACUUAUUAUGGAUCGGA 3'  score=0.5  range=1586-1606
Bd1-1  TCONS_11618.1:miR5181b-5p       5' CGACACUUUUUAUGGAUCGGA 3'  score=2    range=235-255
Bd1-1  TCONS_16601.1:miR5181b-5p       5' CGACACUUUUUAUGGAUCGGA 3'  score=2    range=950-970
Bd1-1  TCONS_1917.1:miR5181b-5p        5' AGACAGUUAUUAUGGAUCGGA 3'  score=2    range=1367-1387
Bd1-1  TCONS_20332.2:miR5181b-5p       5' CGACACUUAUUAUGGAUCAGA 3'  score=2    range=1342-1362
Bd1-1  TCONS_20707.1:miR5181b-5p       5' CGACACUUUUUAUGGAUCGGA 3'  score=2    range=3396-3416
Bd1-1  TCONS_20978.1:miR5181b-5p       5' CGACACUUAUUACGGAUCGGA 3'  score=2    range=1202-1222
Bd1-1  TCONS_25951.1:miR5181b-5p      5' AGACACUUAUUAUGGAUCGGA 3'  score=1    range=324-344
Bd1-1  TCONS_26457.1:miR5181b-5p       5' CGAUACUUAUUAUGGAUUGGA 3'  score=1.5  range=356-376
Bd1-1  TCONS_28045.1:miR5181b-5p       5' CGACACUUUUUAUGGAUCGGA 3'  score=2    range=1153-1173
Bd1-1  TCONS_2857.1:miR5181b-5p        5' AGACAAUUAUUAUGGAUCGGA 3'  score=2    range=1024-1044
Bd1-1  TCONS_29039.1:miR5181b-5p       5' AGACACUUAUUAUGGAUUGGA 3'  score=2    range=1275-1295
Bd1-1  TCONS_30807.1:miR5181b-5p       5' CAACACUUAUUAUGGAUCGGA 3'  score=1    range=26-46
Bd1-1  TCONS_31602.1:miR5181b-5p       5' AGAGACUUAUUAUGGAUCGGA 3'  score=2    range=716-736
Bd1-1  TCONS_34310.1:miR5181b-5p       5' CGACACUUAUUAUGUAUCGGA 3'  score=2    range=420-440
Bd1-1  TCONS_36973.1:miR5181b-5p       5' CGACACUUAUUAUGGAUCGGA 3'  score=0    range=192-212
Bd1-1  TCONS_37031.1:miR5181b-5p       5' CGACACUUAUUAUGGAUCGGA 3'  score=0    range=1142-1162
Bd1-1  TCONS_37506.1:miR5181b-5p       5' UGACACUUAUUAUGGAUCGGA 3'  score=0.5  range=918-938
Bd1-1  TCONS_38080.1:miR5181b-5p       5' UGACACUUAUUAUGGAUCGGA 3'  score=0.5  range=398-418
Bd1-1  TCONS_39530.1:miR5181b-5p       5' AGACACUUAUUAUGGAUCGGA 3'  score=1    range=741-761
Bd1-1  TCONS_41001.1:miR5181b-5p       5' AGACGCUUAUUAUGGAUCGGA 3'  score=1.5  range=252-272
Bd1-1  TCONS_4408.1:miR5181b-5p        5' AGACACUUAUUAUGGAUCGGA 3'  score=1    range=2091-2111
Bd1-1  TCONS_44440.1:miR5181b-5p       5' CGACACUUAUUAUGGAUCGUA 3'  score=2    range=744-764
Bd1-1  TCONS_44546.1:miR5181b-5p       5' CGACAUUUAUUAUGGAUCGGA 3'  score=0.5  range=3-23
Bd1-1  TCONS_45252.1:miR5181b-5p       5' AGACACUUAUUAUGGAUCGGA 3'  score=1    range=771-791
Bd1-1  TCONS_45491.1:miR5181b-5p       5'CGACACUUUAUUAUGGAUCGGA 3'  score=1    range=665-686
Bd1-1  TCONS_46223.1:miR5181b-5p       5' CGACACUUAUUAUGGAUCGGA 3'  score=0    range=297-317
Bd1-1  TCONS_4859.1:miR5181b-5p        5' AGACACUUAUUAUGGAUCGGA 3'  score=1    range=89-109
Bd1-1  TCONS_50121.1:miR5181b-5p       5' UGACAUUUAUUAUGGAUCGGC 3'  score=2    range=628-648
Bd1-1  TCONS_53204.1:miR5181b-5p       5' CGACACUUAUUAUGGAUCGGA 3'  score=0    range=619-639
Bd1-1  TCONS_55843.1:miR5181b-5p       5' AGACACUUAUUAUGGAUCGGA 3'  score=1    range=2103-2123
Bd1-1  TCONS_56816.1:miR5181b-5p       5' AGACAUUUAUUAUGGAUCGGA 3'  score=1.5  range=353-373
Bd1-1  TCONS_59215.1:miR5181b-5p       5' AGACACUUAUUAUGGAUCGGA 3'  score=1    range=3857-3877
Bd1-1  TCONS_59899.1:miR5181b-5p       5' CGACACUUAUUAUGGAUCGGA 3'  score=0    range=948-968
Bd1-1  TCONS_8478.2:miR5181b-5p        5' CGACACUUUUUAUGGAUCGGA 3'  score=2    range=2495-2515
Bd21-3  TCONS_00032583:miR5181b-5p     5' UGACACUUAUUAUGGAUCGGA 3'  score=0.5 range=1258-1278
Bd21-3  TCONS_00004756:miR5181b-5p     5' UGACACUUAUUAUGGAUCGGA 3'  score=0.5  range=195-215
Bd21-3  TCONS_00002931:miR5181b-5p     5'CGGCGACUUAUUAUGGAUCGGA 3'  score=1.5  range=867-888
Bd21  TCONS_00062473:miR5181b-5p       5' CCACACUUAUUAUGGAUCGGA 3'  score=1    range=48-68
Bd21  TCONS_00063331:miR5181b-5p       5' CGACACUUAUUAUGGAUAGGA 3'  score=2    range=175-195
Bd21  TCONS_00069620:miR5181b-5p       5' CGACACUUAUUAUGGAUCGAA 3'  score=2    range=1856-1876
Bd21  TCONS_00073536:miR5181b-5p       5' CGACAAUUAUUAUGGAUCGGA 3'  score=1    range=1007-1027
Bd21  TCONS_00073663:miR5181b-5p       5' CGACACUUUUUAUGGAUCGGA 3'  score=2    range=347-367
Bd21  TCONS_00041905:miR5181b-5p       5' UGACACUUAUUAUGGAUCGGA 3'  score=0.5  range=1417-1437
Bd21  TCONS_00041906:miR5181b-5p       5' UGACACUUAUUAUGGAUCGGA 3'  score=0.5  range=976-996
Bd21  TCONS_00046496:miR5181b-5p       5' AGACACUUAUUAUGGAUCGGA 3'  score=1    range=752-772
Bd21  TCONS_00025716:miR5181b-5p       5' CAACAGUUAUUAUGGAUCGGA 3'  score=2    range=1258-1278
Bd21  TCONS_00013281:miR5181b-5p       5' AGACACUUAUUAUGGAUCGGA 3'  score=1    range=1289-1309
Bd21  TCONS_00006100:miR5181b-5p       5' UGACACUUAUUAUGGAUCGGA 3'  score=0.5  range=356-376
Bd21  TCONS_00003733:miR5181b-5p       5'CGGCGACUUAUUAUGGAUCGGA 3'  score=1.5  range=867-888
Bd21  TCONS_00003853:miR5181b-5p       5' CGACACUUAUUAUGGAUCGGA 3'  score=0    range=821-841
                                          :::::::::::::::::::::
                 miR5181b-5p           3' GCUGUGAAUAAUACCUAGCCU   5'
                 miR5181c-5p           3'   UGUGAAUAACACCUGGCCUCC 5'
                                            ::::::: ::::::.::::::
Bd21  TCONS_00030937:miR5181c-5p       5'   ACACUUACUGUGGAUCGGAGG 3'  score=2  range=307-327
```

b

```
              miR5181a-5p               GGACUGUGAAUAAUACCUAGU
                                        :::::::::::::::::::::
Bd1-1   TCONS_6513.1:miR5181a-5p     5' CCUGACACUUAUUAUGGAUCA 3'  score=0    range=197-217
Bd1-1   TCONS_38080.1:miR5181a-5p    5' CCUGACACUUAUUAUGGAUCG 3'  score=0.5  range=396-416
Bd1-1   TCONS_61917.1:miR5181a-5p    5' UCGGACACUUAUUAUGGAUCG 3'  score=2    range=752-772
Bd1-1   TCONS_1687.1:miR5181a-5p     5' UCCGACACUUAUUAUGGAUCA 3'  score=1.5  range=10-30
Bd1-1   TCONS_8151.1:miR5181a-5p     5' CAAGACACUUAUUAUGGAUCA 3'  score=2    range=981-1001
Bd21-3  TCONS_00053248:miR5181a-5p   5' CAAGACACUUAUUAUGGAUCA 3'  score=2    range=611-631
```

c

```
Bd1-1   TCONS_43318.1:miR5181a-3p    5' UCU-AUCCAUAAUAAGUGUCG 3'  score=2   range=564-583
Bd1-1   TCONS_45252.1:miR5181a-3p    5' UCUGAUCCAUAAUAAGUGUCC 3'  score=2   range=709-729
Bd1-1   TCONS_46223.1:miR5181a-3p    5' UCCGAUCCAUAAUAAGUGUCG 3'  score=2   range=246-266
Bd1-1   TCONS_3169.1:miR5181a-3p     5' UCUGAUCCAUAAUAAGUGCCU 3'  score=1   range=426-446
Bd1-1   TCONS_32567.1:miR5181a-3p    5' UCUGAUCCAUAAUAAGUGUCU 3'  score=2   range=278-298
Bd1-1   TCONS_47981.1:miR5181a-3p    5' UCAGAUCCAUAAUAAGUGUCG 3'  score=2   range=6-26
Bd1-1   TCONS_7889.1:miR5181a-3p     5' UCUGAUCCAUAAUAAGUGUCU 3'  score=2   range=4354-4374
Bd1-1   TCONS_9467.1:miR5181a-3p     5' UCCGAUCCAUAAUAAGUGUCG 3'  score=2   range=2181-2201
Bd21    TCONS_00015127:miR5181a-3p   5' UCCGAUCCAUAAUAAGUGUCG 3'  score=2   range=513-533
Bd21    TCONS_00003853:miR5181a-3p   5' UC-GAUCCAUAAUAAGUGUCG 3'  score=2   range=780-799
Bd21    TCONS_00046496:miR5181a-3p   5' UCUGAUCCAUAAUAAGUGUCC 3'  score=2   range=690-710
                                        ::::::::::::::::::::.:
                 miR5181a-3p     3'     AGACUAGGUAUUAUUCACGGC 5'
                 miR5181d-3p     3' AGGGAGGCCAGGUAUUAUUCA     5'
                                    :::::::: ::::::::::::
Bd21    TCONS_00048303:miR5181d-3p  5' UCCCUCCGAUCCAUAAUAAGU 3'  score=2  range=801-821
Bd21    TCONS_00013281:miR5181d-3p  5' UCCCUCCGGUCCAUAAUAAUU 3'  score=2  range=1222-1242
Bd21    TCONS_00017033:miR5181d-3p  5' UCCCUCCGAUCCAUAAUAAGU 3'  score=2  range=465-485
Bd21-3  TCONS_00037547:miR5181d-3p  5' UCCCUCCGAUCCAUAAUAAGU 3'  score=2  range=801-821
Bd1-1   TCONS_9467.1:miR5181d-3p    5' UCCCUCCGAUCCAUAAUAAGU 3'  score=2  range=2177-2197
Bd1-1   TCONS_9574.1:miR5181d-3p    5' UCCCUCCGAUCCAUAAUAAGU 3'  score=2  range=5752-5772
Bd1-1   TCONS_958.1:miR5181d-3p     5' UCCCUCCGAUCCAUAAUAAGU 3'  score=2  range=292-312
Bd1-1   TCONS_60289.1:miR5181d-3p   5' UCCCUCCGAUCCAUAAUAAGU 3'  score=2  range=540-560
Bd1-1   TCONS_7100.1:miR5181d-3p    5' UCCCUCCGGUCCAUAAUAAGU 3'  score=0  range=1089-1109
Bd1-1   TCONS_59684.1:miR5181d-3p   5' UCCCUCCGAUCCAUAAUAAGU 3'  score=2  range=96-116
Bd1-1   TCONS_59215.1:miR5181d-3p   5' UCCCUCCGAUCCAUAAUAAGU 3'  score=2  range=3791-3811
Bd1-1   TCONS_52520.1:miR5181d-3p   5' UCCCUCCGAUCCAUAAUAAGU 3'  score=2  range=1500-1520
Bd1-1   TCONS_48757.1:miR5181d-3p   5' UCCCUCCGAUCCAUAAUAAGU 3'  score=2  range=2-22
Bd1-1   TCONS_49199.1:miR5181d-3p   5' UCCCUCCGAUCCAUAAUAAGU 3'  score=2  range=336-356
Bd1-1   TCONS_45722.1:miR5181d-3p   5' UCCCUCCGAUCCAUAAUAAGU 3'  score=2  range=3207-3227
Bd1-1   TCONS_46223.1:miR5181d-3p   5' UCCCUCCGAUCCAUAAUAAGU 3'  score=2  range=242-262
Bd1-1   TCONS_4409.1:miR5181d-3p    5' UCCCUCCGAUCCAUAAUAAGU 3'  score=2  range=2997-3017
Bd1-1   TCONS_4408.1:miR5181d-3p    5' UCCCUCCGGUCCAUAAUAAUU 3'  score=2  range=2024-2044
Bd1-1   TCONS_37505.1:miR5181d-3p   5' UCCCUCCGAUCCAUAAUAAGU 3'  score=2  range=1063-1083
Bd1-1   TCONS_35672.1:miR5181d-3p   5' UCCCUCCGAUCCAUAAUAAGU 3'  score=2  range=1177-1197
Bd1-1   TCONS_36973.1:miR5181d-3p   5' UCCCUCCGAUCCAUAAUAAGU 3'  score=2  range=151-171
Bd1-1   TCONS_33191.1:miR5181d-3p   5' UCCCUCCGAUCCAUAAUAAGU 3'  score=2  range=259-279
Bd1-1   TCONS_10832.1:miR5181d-3p   5' UCCCUCCGAUCCAUAAUAAGU 3'  score=2  range=606-626
Bd1-1   TCONS_21020.1:miR5181d-3p   5' UCCCUCCGAUCCAUAAUAAGU 3'  score=2  range=1775-1795
Bd1-1   TCONS_2214.1:miR5181d-3p    5' UCCCUCCGAUCCAUAAUAAGU 3'  score=2  range=2551-2571
Bd1-1   TCONS_23406.1:miR5181d-3p   5' UCCCUCCGAUCCAUAAUAAGU 3'  score=2  range=295-315
Bd1-1   TCONS_25951.1:miR5181d-3p   5' UCCCUCCGAUCCAUAAUAAGU 3'  score=2  range=257-277
Bd1-1   TCONS_27173.1:miR5181d-3p   5' UCCCUCCGAUCCAUAAUAAGU 3'  score=2  range=2974-2994
Bd1-1   TCONS_2857.1:miR5181d-3p    5' UCCCUCCGAUCCAUAAUAAGU 3'  score=2  range=899-919
Bd1-1   TCONS_29039.1:miR5181d-3p   5' UCCCUCCGAUCCAUAAUAAGU 3'  score=2  range=1209-1229
```

The samples included different tissues, organs and stress condition in three Bd inbred lines: Bd21, Bd21-3 and Bd1-1. Library read length ranged from 35 to 150 bp for a total of about 1.9 billion raw reads

Samples	Inbred line	SRA accession	Experiment	Project	References
Leaf 20 Day **	Bd21	SRR349785; SRR352143	Poly (A) RNA-Seq	Brachypodium distachyon, Bd21 RNA-Seq - Conserved Poaceae Specific Genes Project	Davidson et al., 2012
Emerging inflorescence	Bd21	SRR349787	Poly (A) RNA-Seq	Brachypodium distachyon, Bd21 RNA-Seq - Conserved Poaceae Specific Genes Project	Davidson et al., 2012
Early inflorescence	Bd21	SRR349786	Poly (A) RNA-Seq	Brachypodium distachyon, Bd21 RNA-Seq - Conserved Poaceae Specific Genes Project	Davidson et al., 2012
Anther	Bd21	SRR352140	Poly (A) RNA-Seq	Brachypodium distachyon, Bd21 RNA-Seq - Conserved Poaceae Specific Genes Project	Davidson et al., 2012
Pistil	Bd21	SRR352137	Poly (A) RNA-Seq	Brachypodium distachyon, Bd21 RNA-Seq - Conserved Poaceae Specific Genes Project	Davidson et al., 2012
Seed 5 DAP	Bd21	SRR352139	Poly (A) RNA-Seq	Brachypodium distachyon, Bd21 RNA-Seq - Conserved Poaceae Specific Genes Project	Davidson et al., 2012
Seed 10 DAP	Bd21	SRR352141	Poly (A) RNA-Seq	Brachypodium distachyon, Bd21 RNA-Seq - Conserved Poaceae Specific Genes Project	Davidson et al., 2012
Embryo 25 DAP **	Bd21	SRR352138; SRR352144	Poly (A) RNA-Seq	Brachypodium distachyon, Bd21 RNA-Seq - Conserved Poaceae Specific Genes Project	Davidson et al., 2012
Endosperm 25 DAP	Bd21	SRR352142	Poly (A) RNA-Seq	Brachypodium distachyon, Bd21 RNA-Seq - Conserved Poaceae Specific Genes Project	Davidson et al., 2012
Third leaf	Bd21	SRR5645253	Poly (A) RNA-Seq	Brachypodium distachyion, Bd21 RNA-seq - Thrid leaf Transcriptome Project	Bertolini et al., Unpublished
Several tissues and treatments§	Bd21	-	EST	Full-length enriched Brachypodium distachyon cDNA library	Mochida et al., 2013
Shoots Mock-inoculated	Bd21-3	SRR1635409	Poly (A) RNA-Seq	Brachypodium distachyon Transcriptome or Gene expression	Mandadi et al., 2015
Shoots PMV-infected	Bd21-3	SRR1635416	Poly (A) RNA-Seq	Brachypodium distachyon Transcriptome or Gene expression	Mandadi et al., 2015
Shoots PMV+SPMV-infected	Bd21-3	SRR1635430	Poly (A) RNA-Seq	Brachypodium distachyon Transcriptome or Gene expression	Mandadi et al., 2015
Stem Parenchyma Tissue	Bd21-3	SRR1797573; SRR1797574; SRR1797575; SRR1797576; SRR1797577; SRR1797578; SRR1797579; SRR1797580; SRR1797581; SRR1797582	Poly (A) RNA-Seq	Brachypodium distachyon strain:Bd21-3 Transcriptome or Gene expression	Unpublished
Leaf	Bd1-1	SRR1425915	Poly (A) RNA-Seq	Brachypodium distachyon Transcriptome or Gene expression	Gordon et al., 2014

Supplemental Table 1. Description of data set used to identify lncRNAs.

(**) indicates two biological replicates.

(§) Tissues and treatments present in the cDNA library (seed at germination, leaf at vegetative stage and after flowering, shoot, crown, spikes at flowering and at different stages after pollination, callus and leaf at 2 weeks after germination treated with different stresses and compounds)

Samples	Raw Reads	Reads Trimmed	% Reads Trimmed	Mapped Reads	% Mapped Reads
Leaf 20 Day **	56673394	52637592	92.88%	51686304	98.19%
Emerging inflorescence	23137165	21880389	94.57%	21315167	97.42%
Early inflorescence	17601221	16771555	95.29%	16191851	96.54%
Anther	26059840	23226658	89.13%	22380996	96.36%
Pistil	17712829	16739912	94.51%	15759963	94.15%
Seed 5 DAP	25922555	22737023	87.71%	22094542	97.17%
Seed 10 DAP	25766517	24540576	95.24%	21938177	89.40%
Embryo 25 DAP **	48681058	44944894	92.33%	42649788	94.89%
Endosperm 25 DAP	27365511	24533485	89.65%	23314366	95.03%
Third leaf	436558137	406485650	93.11%	397,120,116	97.70%
Shoots Mock-inoculated	20252166	18636574	92.02%	17911661	96.11%
Shoots PMV-infected	18988712	17545219	92.40%	16775620	95.61%
Shoots PMV+SPMV-infected	20570446	19069829	92.70%	18151628	95.19%
Stem Parenchyma Tissue	495586358	380085749	76.69%	340877783	89.68%
Leaf	632286864	456101227	72.14%	363,676,588	79.74%
**Total**	**1893162773**	**1545936332**	**81.66%**	**1391844550**	**90.03%**

Supplemental Table 2. Description of the alignment statistics.

Mapped reads refers to the results of the second iteration of mapping process. (**) indicates two biological replicates.

Supplemental Document 1

In order to validate lncRNAs identified by our bioinformatic pipeline, we performed RT-PCR amplifying nine lncRNAs expressed with different RPKM values in six Bd21 tissues (third leaf, leaves 20 day, early inflorescence, emerging inflorescence, seed 5 DAP and seed 10 DAP). Each tissue was collected and grinded with mortar and pestle, successively the total RNA was extracted using Plant/Fungi Total RNA Purification Kit (Norgen Biotek) and the RNA extracted was subjected to a DNase treatment using RNase-Free DNase I Kit (Norgen Biotek). cDNA was retrotranscribed from 1 μg of total RNA using iScript™ cDNA Synthesis Kit (Biorad) following the manufacturer's instructions. We amplified lncRNAs by RT-PCR characterized by 60.5 ℃ of annealing temperature and 30 amplification cycles. *Actin* (Bradi4g41850) were chosen as housekeeping and were amplified in each tissue considered. Finally the length of amplified product were checked with an agarose gel (Figure 1). Table 1 lists the information about primers used to perform RT-PCR.

Primer Name	Sequence	Amplified Sequence Length	Tissue
TCONS_00032695_F	CCAGCGAAGATCAAAGCAACAG	154	Third Leaf
TCONS_00032695_R	GCTGGAAAGGTAAGAGGGACAG		
TCONS_00057000_F	CGATCGCACAAACTGGACCT	126	Third Leaf
TCONS_00057000_R	GCCAAATCCCTGCTACTCAC		
TCONS_00031368_F	GTTGGTGGTTGCGAGTGAAAG	234	Third Leaf
TCONS_00031368_R	CCCGACAAAGGTTTCAAAGGC		
TCONS_00028489_F	ACAACAGCCCCTTCCACATG	166	Leaves 20 Days
TCONS_00028489_R	CCTTTCCCATTCGCCGATCA		
TCONS_00065961_F	AAGCCTTTCCCCTTCCCTCT	242	Early Inflorescence
TCONS_00065961_R	AGTGATTCTTCCGCCCATCA		
TCONS_00069782_F	ATGAAGGTGGTGGTCGTTGTT	243	Emerging Inflorescence
TCONS_00069782_R	TTATACCGGCAGCAGTGTAGC		
TCONS_00005342_F	GTCACGCGATGTTGTGATCCT	163	Seed 5 DAP
TCONS_00005342_R	TGACTGGTCGCTATCACGGT		
TCONS_00004579_F	CCATCTCAGGGTCTCGCGTAA	166	Seed 10 DAP
TCONS_00004579_R	CTCTCAGTCTCCCCGATCTCA		
TCONS_00027464_F	AATCCCTCCCGACGTGCAAT	216	Seed 10 DAP
TCONS_00027464_R	TGACAGACTGGTTCAAGGCGT		
Bradi4g41850_F	GTGAGTATGATGAGTCTGGTCCAG	151	
Bradi4g41850_R	TACGAGTCTAGGAGGTACACAG		

Table 1. List of primers used to amplify lncRNAs with RT-PCR.

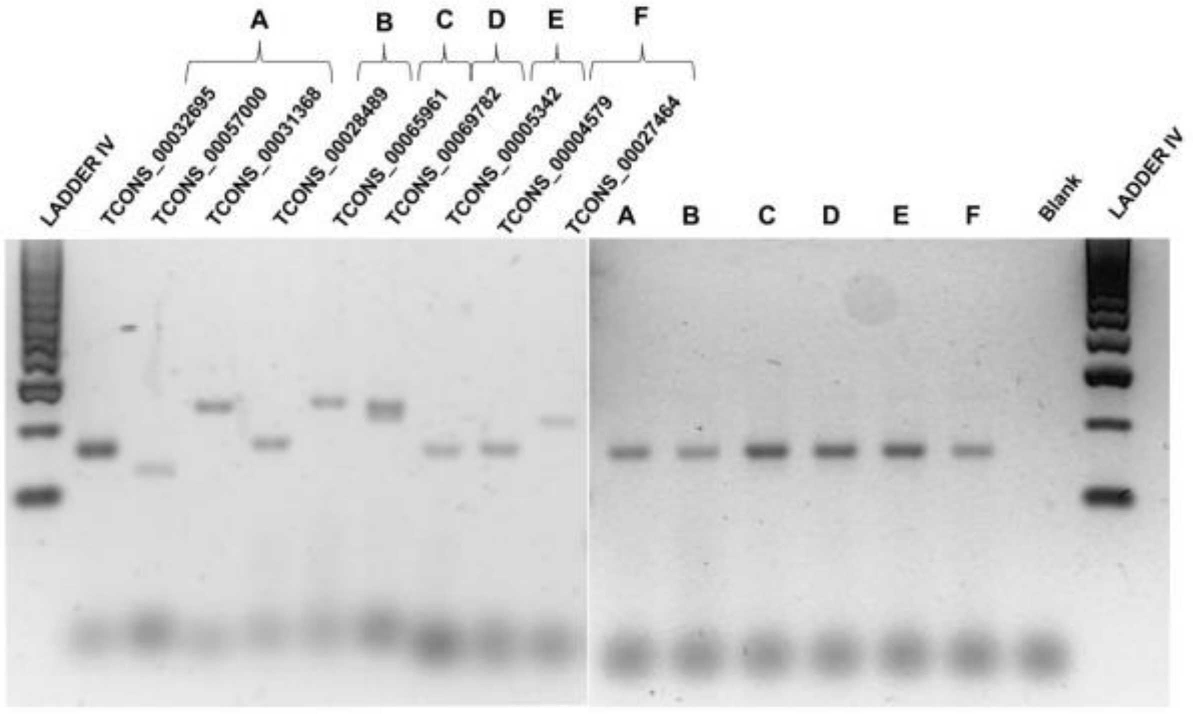

Figure 1. Agarose gel of RT-PCR of lncRNAs *in silico* predicted in six Bd21 tissues.

The left panel shows the lncRNAs amplified products. The right panel shows the amplification of *Actin* in each Bd21 tissue. Letters indicate Bd21 tissues: (A) Third leaf, (B) Leaves 20 day, (C) Early inflorescence, (D) Emerging inflorescence, (E) Seed 5 DAP, (F) Seed 10 DAP. We used as marker of band size HyperLadder IV.
